# Site‐Specific Drivers of Land‐Use Change Effects on Organic Carbon in German Agriculture and Forest Soils

**DOI:** 10.1111/gcb.70576

**Published:** 2025-10-28

**Authors:** David Emde, Ali Sakhaee, Christopher Poeplau, Axel Don, Marc Scherstjanoi, Nicole Wellbrock, Florian Schneider

**Affiliations:** ^1^ Johann Heinrich von Thünen Institute, Institute of Climate Smart Agriculture Braunschweig Germany; ^2^ Johann Heinrich von Thünen Institute, Institute of Forest Ecosystems Eberswalde Germany

**Keywords:** agriculture, forest, land‐use change, LUC, LULUCF, SOC, SOC drivers, soil carbon, soil depth, subsoil

## Abstract

Understanding the extent to which land‐use changes (LUC) impact soil organic carbon (SOC) is essential for accurate carbon accounting and global efforts aimed at reducing the negative impact of LUC on climate change. Recognizing that most of the SOC change due to LUC occurs in the topsoil, current efforts to quantify SOC change often overlook subsoils beyond 30 cm depth. We used data from Germany's national agricultural and forest soil inventories to address this sparsity by modeling depth‐dependent, LUC‐induced, SOC stock change down to 90 cm using data‐driven reciprocal modeling. This modeling was carried out using an ensemble approach for prediction and area of applicability assessments to avoid extrapolation. Landscape, climate, and pedological properties were used to predict the equilibrium SOC stock at four depth intervals (0–10, 10–30, 30–60, and 60–90 cm) for all six land‐use change directions between cropland, grassland, and forest. While the greatest change occurred at the surface for all LUC directions, we detected significant SOC stock change down to the sampled depth of 90 cm. Approximately 30% of the detected SOC stock change was found in the subsoil (30–90 cm). For LUC to or from forests, the litter layer dominated the changes in SOC such that for LUC between grassland and forest, SOC stock change in the mineral soil was mostly offset by the addition or removal of the litter layer. For all LUC directions, the World Reference Base soil group was the most important factor for determining the magnitude of SOC stock change. This study underscores the importance of deeper soil sampling for accurate carbon accounting and climate‐change mitigation strategies.

## Graphical abstract

Land‐use change—for example, turning cropland into forest—alters soil organic carbon (SOC) storage to different degrees depending on soil type. Data from the German national agricultural and forest soil inventories and new modeling techniques show that, while most of this SOC change occurs in the topsoil, 30% occurs below 30 cm depth. These insights can help improve agricultural carbon accounting and emissions reporting, aiding in global efforts to mitigate climate change.

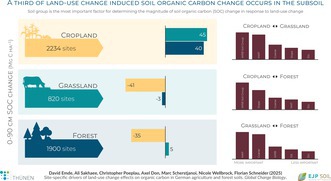

## 1 Introduction

Efforts aimed at reducing the impact of agricultural systems on climate change often encourage transitioning land to a more natural state—either for a few years at a time (as with ley rotations) or permanently (Buendia et al. 2019; Federal Environmental Agency 2021). Such efforts not only aim to limit soil organic carbon (SOC) loss but also recognize the importance of SOC to sustainable agricultural production (Mattila and Vihanto 2024; Rabot et al. 2024; Romero et al. 2024). Changing land from one use to another brings with it changes to plant species, management, and disturbance regimes (Emde et al. 2024; L. B. Guo and Gifford 2002; Sanderman et al. 2017). Consequently, the soil characteristics and processes responsible for C cycling are altered. Understanding how these alterations interact with site properties is therefore important for properly accounting for SOC changes, limiting losses that could aggravate climate change, and maintaining healthy soils capable of keeping up with increasing global demand for food (Wiesmeier et al. 2016).

While the litter layer accounts for a large portion of SOC stocks in forest systems, roots are the interface between plants and mineral soil (Grüneberg et al. 2019). As such, roots are direct (root turnover and exudates; Fitter et al. 1997; Poeplau et al. 2021) or indirect (root channels, soil aggregation, improved hydrological properties, interactions with microbes; Ma et al. 2023; Poeplau et al. 2021; Souza et al. 2023) drivers of many important soil processes. Because roots are most dense in the topsoil (typically 0–30 cm), and in the first 10 to 20 cm in particular, changes to SOC following any sort of disturbance—like changes to plant and management following LUC—tend to be greatest near the surface and decrease with depth (Jobbágy and Jackson 2000; McGranahan et al. 2014). When combined with the difficulty of sampling beyond the topsoil depth, it appears understandable that most of the current literature on SOC change following LUC focuses only on the top 20 or 30 cm of the soil profile (Grüneberg et al. 2014; Huang et al. 2024; Poeplau et al. 2011; Zhang et al. 2024). While there are a few studies that note SOC changes below topsoil depth following LUC, they often either rely on very limited data or measure SOC pre‐ and post‐LUC at too short an interval to ensure SOC equilibrium with regard to LUC. For example, Liu et al. (Liu et al. 2018) found subsoil effects in pasture to cropland conversion in temperate areas of China (−16% loss), but only measured subsoil to 60 cm depth on a site that had been cropland for 27 years. Meanwhile, Li et al. (2018) reported SOC stock increases to 1 m as a single depth increment (42 C Mg ha^−1^) in global sites converted from cropland to grassland, thereby obfuscating the depth effect of LUC.

The current literature increasingly supports the notion of a centennial timescale to LUC effects on SOC (Emde et al. 2024; Li et al. 2018; Springob et al. 2001). For example, Emde et al. (2024) and Li et al. (2018) both estimated that it takes nearly a century to reach SOC equilibrium with regard to LUC when croplands are converted to grasslands. For grassland to cropland LUC, Emde et al. (2024) found that SOC equilibrium wasn't reached for closer to two centuries, while Springob et al. (2001) found that it takes about 100 years. In any case, the decades it takes to reach new SOC equilibrium following LUC means it is often necessary to compare separate sites under different land uses but with similar landscape, soil, and climatic characteristics rather than resampling the same site through time. Although this paired‐site (space for time) approach solves the issue of time between samplings, it does have potential shortcomings. First, the sites with different land uses can only be paired based on a limited set of criteria and site‐specific differences in SOC change dynamics may, as such, unintentionally be omitted. Secondly, assumptions related to the equilibrium status of each site may lead to comparing sites not at equilibrium. Where repeat samplings are able to take place at the same site through time, the time since LUC is often limited (as with the example from Li et al. (2018) above) and such investigations likely do not capture equilibrium to equilibrium SOC change.

Working with extensive soil, landscape, climatic, and land‐use history data from the German National Agricultural (Poeplau et al. 2020) and Forest Soil Inventories (Wellbrock et al. 2019), we used data‐driven reciprocal modeling to examine site‐specific potential SOC change at depth following LUC (Schneider et al. 2021). We used data‐driven reciprocal modeling to estimate SOC stock change between cropland, grassland, and forest sites at two topsoil depths (0–10 cm and 10–30 cm) and two subsoil depths (30–60 cm and 60–90 cm) in order to answer the following questions: (i) how much does SOC stock change at all depths following LUC between cropland, grassland, and forest (6 LUC directions and 4 depths), (ii) to which depth are LUC effects on SOC detectable, (iii) how much of the LUC effect occurs in the subsoil, and (iv) which soil, landscape, and climatic factors affect the magnitude of SOC change?

## 2 Methods

### 2.1 Soil and Site Data

Data used in this study were gathered during two national German soil inventories: the German Agricultural Soil Inventory (Bodenzustandserhebung Landwirtschaft, carried out from 2011 to 2018; Poeplau et al. 2020) and the National Forest Soil Inventory (Bodenzustandserhebung im Wald II, carried out from 2006 to 2008; Wellbrock et al. 2006, 2022). Both inventories were sampled via the same 8 × 8 km^2^ grid across Germany and used the same ISO Standards for soil physicochemical analysis (GAFA 2014). Agricultural soils were sampled at five fixed‐depth increments from one profile pit (0–10 cm, 10–30 cm, 30–50 cm, 50–70 cm, and 70–100 cm). Samples were taken for forest soils at five depth increments (0–5 cm, 5–10 cm, 10–30 cm, 30–60 cm, 60–90 cm) from both profile pits and eight mixed satellite cores of known volume and size appropriate to the site conditions. Multiple forest litter fractions were collected using approximately 20 × 20 cm frames and mixed satellite samples. To avoid the confounding effects of seasonality on forest litter accumulation, the litter stocks reported here are composed of the litter fractions that remain relatively stable between seasons: wood and fruit litter, moderately decomposed litter (Of), and highly decomposed (Oh) fractions smaller than 2 mm, and litter < 20 mm.

**FIGURE 1:**
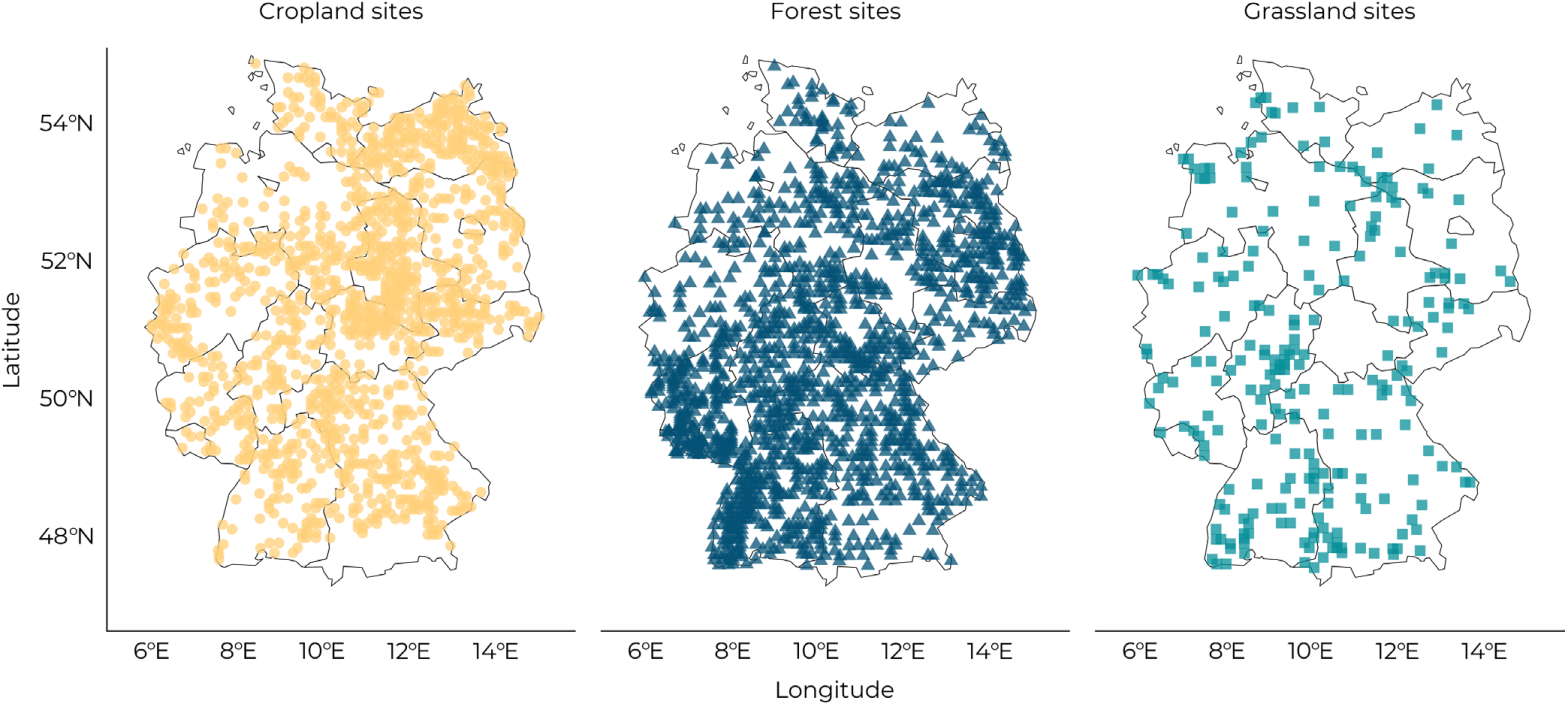
Locations of sites under all three land uses covered in this study.

The Agricultural Soil Inventory consists of 2234 cropland sites and 820 grassland sites, while the National Forest Soil Inventory consists of 1900 forest sites (Figure 1). Agriculture in Germany covers nearly half of the land area nationwide, the majority of which is annual cropland (70% of agricultural area) and grassland (28% of agricultural area; Destatis 2019). Cereals (wheat and barley) and canola are the most common annual crops in Germany (FAOSTAT 2022), and grasslands are predominantly intensively managed pastures (58% pasture, 42% meadow; Destatis 2019). Forests, meanwhile, cover about a third of the land area in Germany and largely consist of pine (22%) and spruce (21%) trees (*Bundeswaldinventur—Ergebnisdatenbank* 2024). Forests in Germany are managed systems and, in most cases, have been re‐planted over the past decades and centuries. Soil texture ranged from very clayey (76% clay) to very sandy (96% sand) across all sites.

Soil properties for the German Agricultural Soil Inventory were measured at the Thünen Institute, Germany; the details of which are outlined in Poeplau et al. (2020). All assessments and analyses for the National Forest Soil Inventory were carried out by the federal states alongside an extensive system of quality assurance measures—including comparability assessments between soil profile and soil core sampling. Details regarding specific laboratory analysis and data collection can be found in Wellbrock et al. (2019). In brief, soil texture (clay, < 2 μm; silt, 2–63 μm; and sand, 63–2000 μm content), bulk density, and rock fragment fraction were measured prior to total C and total N elemental analysis (dry combustion). Ramped combustion was used to separate organic C (volatilization at < 550°C) from inorganic C (volatilization between 550°C and approximately 1000°C).

Soil type according to the German system of soil classification was assessed at the time of sampling by field surveyors and converted to World Reference Base (WRB) soil group for the purposes of this study. Cambisols (39%), Luvisols (13%), and Stagnosols (13%) comprised a majority of the soil groups for the included sites. Climate variables and terrain characteristics were collected using specific site coordinates alongside open data sources. Drought index, mean annual precipitation, and mean annual temperature (all 30‐year averages) were collected from the German Meteorological Service's open data server (Deutscher Wetterdienst 2022), and landscape data (elevation and slope) were derived from EcoDataCube (Witjes et al. 2023). Soil horizons were classified according to Ad‐hoc Sponagel (2005) by trained field surveyors at soil pits. Sites included in this study had an average mean annual precipitation of 803 mm (491 to 2144 mm) and an average mean annual temperature of 8.9°C (4.6°C to 11.3°C).

Soil organic carbon stock (Mg C ha^−1^) was calculated as follows:

(1) $$\mathrm{SO}C_{\text{stock}}=\mathrm{SO}C_{\mathrm{con}}*BD_{\text{fine soil}}*\left(1-\text{rock fragment fraction}\right)*\text{depth}$$

where $\mathrm{SO}C_{\mathrm{con}}$ is the SOC content in the fine soil < 2 mm in %, $BD_{\text{fine soil}}$ is the bulk density of the fine soil in g cm ^−3^, rock fragment fraction is the volumetric fraction of particles > 2 mm in vol%/100, and depth is the thickness of the depth increment in cm. Rock fragment fraction was determined for both inventories using measurements from soil pits. This approach to SOC stock calculation accounts for differences in coarse content and avoids systematic overestimation of SOC stocks (Poeplau et al. 2017).

Depth increments were made to match between the agricultural and forest inventories by downscaling the agricultural inventory to 1 cm increments, assuming constant values within the sampled depth increments in most cases. Where horizon boundaries split depth increments, downscaling was adjusted to account for SOC stocks on either side of the boundary. Data were then aggregated to the four depth increments used here (0–10 cm, 10–30 cm, 30–60 cm, and 60–90 cm) using weighted means (Figures S1–S5). Weighting factors were either increment thickness (BD, coarse content) or fine soil stock (clay, silt, N, OC, and pH).

### 2.2 Data‐Driven Reciprocal Modeling

**FIGURE 2:**
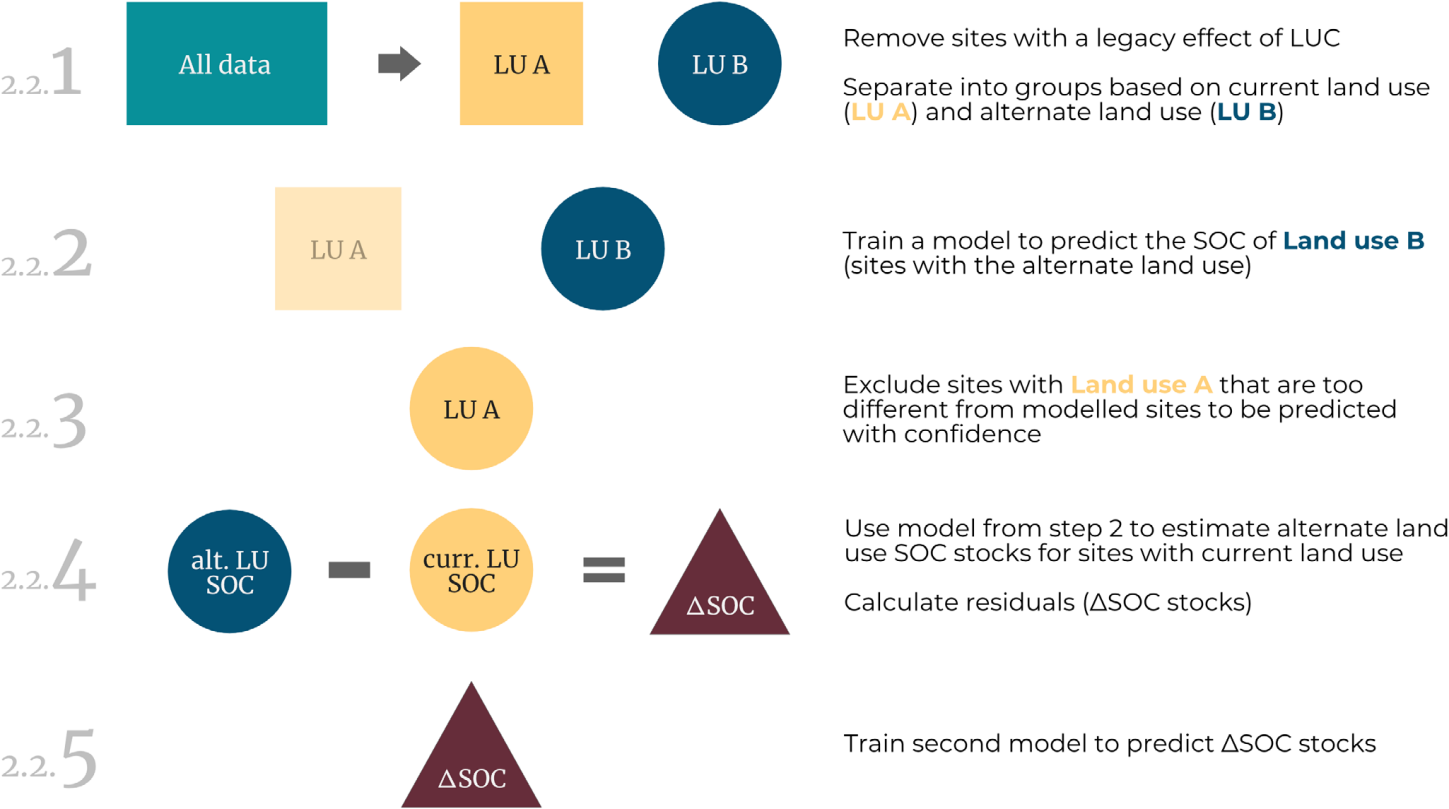
Process outline for the data‐driven reciprocal modeling pipeline as used in this study. Steps in the left margin denote the manuscript section in which each part of the process is detailed.

Data‐driven reciprocal modeling was used to estimate the SOC stock of sites as if they were under a different land use (referred to as “alternate” land use from hereon). This 5‐step pipeline was derived from Schneider et al. (2021) and is also outlined in Figure 2. The following was carried out once for each LUC direction (i.e., cropland to grassland LUC, forest to cropland LUC, etc.) and soil depth increment:

*Step 1*: Separate data into groups by current land use such that one group contains only sites under the current LU, and the second group contains only sites under the alternate LU (e.g., for modeling cropland to grassland LUC, the “current” LU group contains only permanent croplands, and the “alternate” LU group contains only permanent grasslands).

*Step 2*: Train an ensemble model to predict the SOC stock of all sites with the alternate LU using predictor variables that are expected to have an impact on SOC but are not affected by land use (e.g., precipitation, clay content, etc.).

*Step 3*: Compare the site properties of sites with the current LU to those with the alternate LU. Remove sites of the current LU that are too different from those of the alternate LU to be predicted without extrapolation (i.e., outside the area of applicability).

*Step 4*: Use the ensemble model produced in *step 2* (i.e., trained on the sites of the alternate LU) to predict SOC stocks for sites with the current LU that are inside the area of applicability. The residual, calculated as the difference between the predicted and measured SOC stock, represents the change in SOC stock attributable to LUC ($\mathrm{\Delta SO}C_{\text{stock}}$).

*Step 5*: Train a second ensemble model on the $\mathrm{\Delta SO}C_{\text{stock}}$ values calculated in *step 4*. Permutation variable importance for this second model is then used to determine the predictors with the greatest impact on the change in SOC stock following LUC.

#### 2.2.1 Step 1—Data Preparation

To ensure that SOC stocks were representative of mineral soils under permanent land use, included sites were non‐organic soils that, under current and alternate land use, are assumed to be at SOC equilibrium with regard to LUC. Organic soils were excluded on the basis of SOC content. In German agricultural soils, 8.7% is often used as the SOC threshold for organic soils; however, the WRB soil classification system defines the threshold at 20% SOC (IUSS Working Group WRB 2022). Here, in the interest of accuracy to the German context while retaining generalizability, a site was considered to have organic soil, and therefore excluded, if SOC content at any depth increment was greater than 10% or it was a Histosol as per the WRB soil classification system (110 sites). Permanency of land use, and thus SOC equilibrium, was evaluated based on individual site histories as per Emde et al. (2024). Briefly, sites were considered to be at approximate equilibrium if they had undergone no LUC throughout their compiled history or had been under the requisite land use long enough prior to sampling that SOC was no longer changing as a result of historic LUC (151 years under continuous cropland or 79 years under continuous grassland; Emde et al. 2024). Additionally, cropland sites with ley rotations were excluded as they are considered to be under constant flux. Samples of all forest sites were taken prior to large tree die‐offs in recent years and were considered to be at equilibrium with regard to LUC. While it is likely that climate change and management‐related influences on SOC are ongoing despite the permanency of land use, studies using the same dataset have shown that such effects are an order of magnitude smaller than LUC effects in the German context (Poeplau et al. 2020; Poeplau and Dechow 2023). As such, we do not expect management and climate effects to significantly impact our results and do not specifically account for management and climate here. However, it should be noted that the term “equilibrium” used in this study, consequently, relates only to LUC effects on SOC and does not necessarily imply generally stable SOC.

#### 2.2.2 Step 2—Train Predictive Model on SOC of Sites With Alternate Land Use

While the reciprocal modeling pipeline is model‐agnostic, meaning it can incorporate any type of machine learning algorithm or modeling framework, for this study we used a stacked ensemble model based on the R package *mlr3* (Lang et al. 2019) that utilizes random forest (*ranger*; Wright and Ziegler 2017), boosted regression trees (*gbm*; Ridgeway and GBM Developers 2024), and support vector regression (*svm* function from the *e1071* package; Meyer et al. 2023) as the base learners. Each base learner contributed equally to the final value, with outputs combined through simple means. Stacked ensemble modeling is computationally expensive but has the advantage of offsetting individual model type biases by combining the results of multiple models, leading to more robust and accurate generalization (Mienye and Sun 2022). The ensemble model used in this study is outlined in detail in Sakhaee et al. (2024). A 5‐fold spatial cross‐validation (CV) approach was applied to evaluate model performance. Germany was divided into 50 strata using a 100 × 100 km^2^ grid (INSPIRE Maintenance and Implementation Group 2023), and random samples were drawn from each stratum to create each fold for the outer loop of the CV. The spatial CV was repeated ten times to ensure robustness of model evaluation. Within each iteration of the outer loop, the training set was further partitioned into 5 folds, forming a nested CV structure for hyperparameter optimization. A random search algorithm was then applied to optimize the hyperparameters of the models. This framework of nested CV ensures the model evaluation is unbiased (Varma and Simon 2006). The final per‐site SOC stock prediction was obtained by averaging the results from the 10 CV repetitions. Model uncertainty was quantified by calculating the interquartile range (IQR) from these 10 predictions using the IQR function of the *stats* package (Figures S6–S11; R Core Team 2024). Model accuracy was estimated using MAE and RMSE (*mae* and *rmse* functions, respectively, both from the *mlr3* R package; Lang et al. 2019). The MAE for cropland models was on average 4.3 across all depths, while the RMSE was 6.6. The MAE for grassland models was on average 10.8, while the RMSE was 16.0. The MAE for forest models was on average 7.9 across all mineral soil depths, while the RMSE was 13.2. Finally, the litter layer MAE was 12.3, while the RMSE was 17.2 (details in Table S2).

Predictors included in model training were those determined by expert knowledge to not be affected by land use but are likely to have an impact on SOC content (Table S1). For cropland and grassland models, these included predictors related to soil texture (e.g., clay and silt content, coarse fragments > 2 mm), organic matter turnover (e.g., soil pH and C:N ratio), site characteristics (e.g., groundwater level, slope, and elevation), climatic variables (e.g., drought index and mean annual precipitation and temperature), WRB reference soil group, and variables related to pedogenesis and soil horizon composition (e.g., parent material type and horizon symbols). For models including forest land use as either the current or target land use, soil pH (Clarholm and Skyllberg 2013) and C:N ratio (Fleck et al. 2019; Högberg et al. 2021) were excluded because they are directly affected by forest‐specific biotic processes and litter composition; therefore, land use. Because C dynamics in the litter layer are different from those in mineral soil, predictor variables for litter layer models were limited to the soil group, climate variables (as above), and site characteristics (elevation and slope).

#### 2.2.3 Step 3—Assess Area of Applicability

In order to avoid extrapolation—predicting on sites outside the predictor space—we used Area of Applicability (AOA) models (*aoa* package in R; Meyer et al. 2024) to compare the sites from the current and target land‐use groups based on all predictors. Full details regarding this approach can be found in Schneider et al. (2021). In short, each site was represented by a point in an n‐dimensional predictor space, where each dimension is a predictor for the model in *step 2*. The location of each point in this space was, thus, defined by the intersection of all values for each of these predictors. Variables more likely to impact the predicted SOC stock were considered to be more important to decisions regarding similarity of sites for modeling purposes. As such, values were additionally weighted using the permutation importance metrics of each predictor from the model produced in *step 2* (Figures S12–S17). Euclidean distance was then calculated between points with the target land use and the closest sites under the current land use. Points that fell outside the *aoa* function default threshold of 0.95 were considered to be too different from the points with the alternate LU to prevent extrapolation and are, thus, excluded from the following calculations (Meyer et al. 2024; Meyer and Pebesma 2021).

#### 2.2.4 Step 4—Predict SOC for Sites Under New Land Use

To estimate the SOC stock for a site under a different land use, the ensemble model produced during *step 2* (model trained on SOC stocks of sites with alternate land use) is applied to sites with a current land use that is different from the modeled alternate land use. For example, when investigating land‐use change from cropland to grassland, the ensemble model built on the properties and SOC stocks of grassland sites is applied to sites that are currently cropland. The difference between this predicted SOC stock and observed SOC stock is, therefore, the expected change in equilibrium SOC stock if a given site were converted to the alternate land use:

(2) $$\mathrm{\Delta SO}C_{\text{stock}}=\text{predicted}\;\mathrm{SO}C_{\text{stock}}-\text{observed}\;\mathrm{SO}C_{\text{stock}}$$

where all $\mathrm{SO}C_{\text{stock}}$ values are given in Mg ha^−1^. Finally, confidence intervals for the national mean ΔSOC stock of each LUC direction and depth were produced using bootstrapping with 10,000 bootstrapped replicates (*boot* and *boot.ci* packages; Canty and Ripley 2021; Davison and Hinkley 1997).

#### 2.2.5 Step 5—Model Drivers of SOC Change

For each site and LUC direction, one ΔSOC stock value was calculated as the sum of absolute $\mathrm{\Delta SO}C_{\text{stock}}$ across all depths. A second, post hoc, model was then trained to predict this absolute ΔSOC stock to 90 cm depth for each pair of LUC directions (i.e., one model for the magnitude of change from cropland to grassland and grassland to cropland together). This model used the same ensemble modeling approach (including cross‐validation and hyperparameter tuning) and training parameters as the model in *step 2*, but the predictor variables were limited to those with established mechanistic interactions with SOC change to enhance interpretability of the model output (soil texture, climate, groundwater, NDVI, etc.; Supporting Information Table S1). The conditional effect of predictors on ΔSOC stock was determined using accumulated local effects (ALE). Permutation variable importance and ALE were then probed for insights into the site‐specific factors affecting SOC change following land‐use change. Both variable importance and ALE were determined using tools from the *mlr3* package (Lang et al. 2019).

## 3 Results

### 3.1 Change Between Cropland and Grassland

**FIGURE 3:**
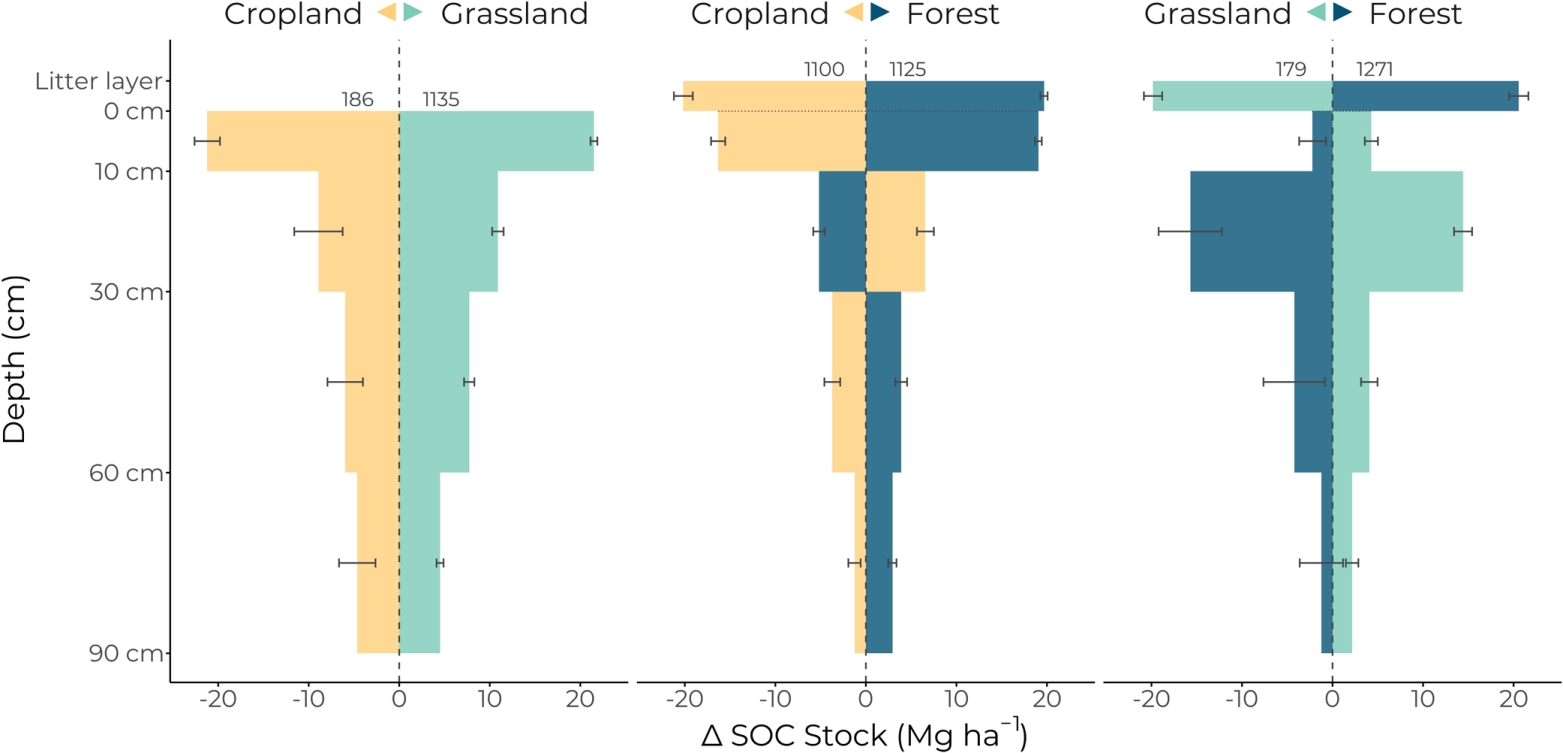
Soil organic carbon change as determined using data‐driven reciprocal modeling of land‐use change (LUC) between all six possible LUC directions between cropland, grassland, and forest. The colored arrow in each facet label denotes the direction of LUC by color (e.g., blue is always land‐use change to forest). Numbers above the bars for each LUC direction indicate the number of sites included in each estimate. The 0‐change line is illustrated with a vertical dashed line such that bars to that extend to the right show positive change, and to the left show negative change. Bootstrapped 95% confidence intervals are shown for each depth and land‐use change direction. Where the confidence interval crosses the 0‐change line, there is no significant change for that depth and direction of LUC.

**FIGURE 4:**
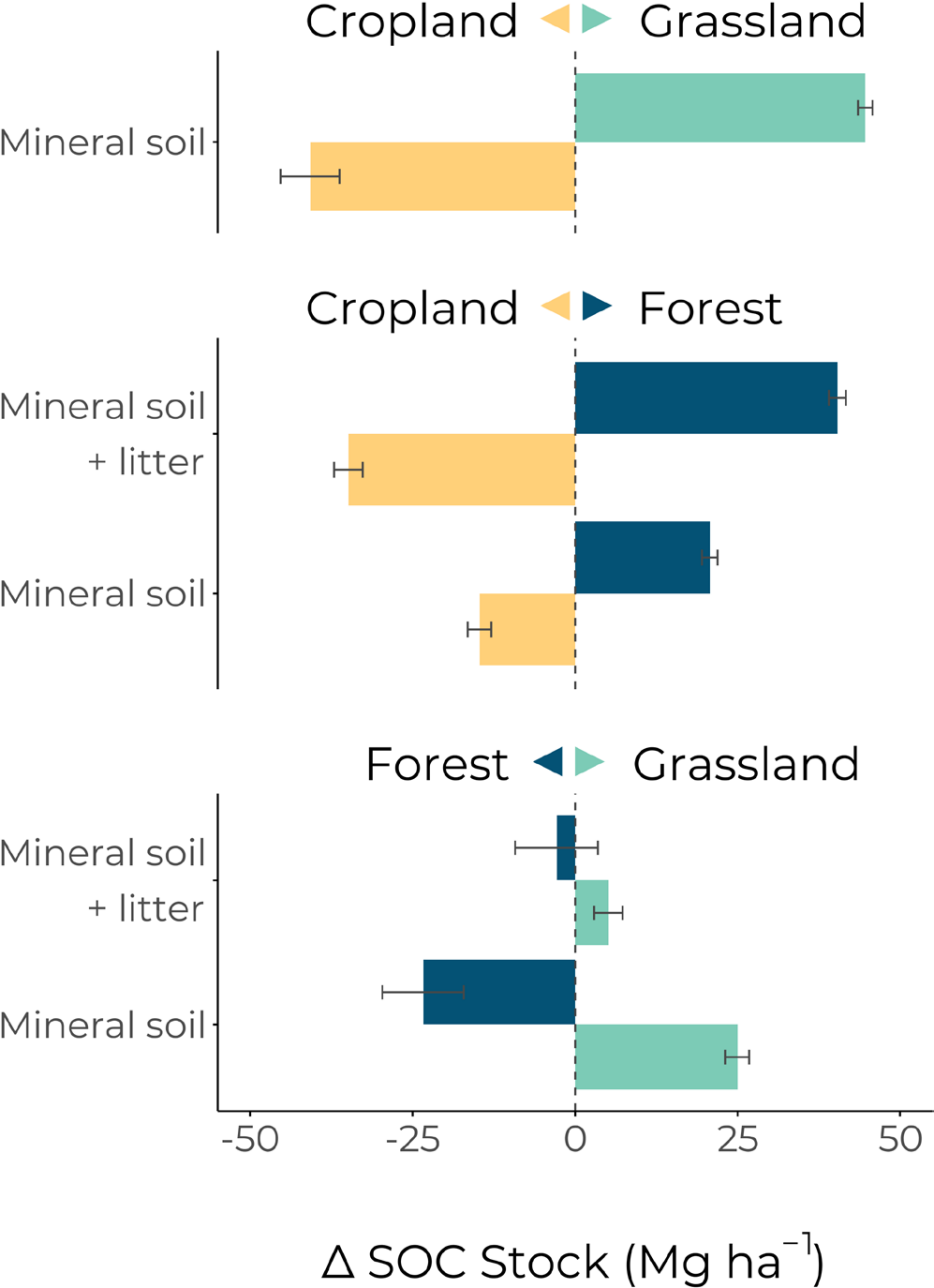
Overall effect of land‐use change (LUC) down to 90 cm soil depth for all six LUC directions between cropland, grassland, and forest. The colored arrow in each facet label denotes the direction of LUC by color (e.g., blue is always land‐use change to forest). The 0‐change line is illustrated with a vertical dashed line such that bars to that extend to the right show positive change, and to the left show negative change. Bootstrapped 95% confidence intervals are shown for each depth and land‐use change direction. Where the confidence interval crosses the 0 change line, there is no significant change for that depth and direction of LUC.

The difference between the predicted SOC stock following LUC from cropland to grassland and vice versa and the current, measured SOC stock was the largest at the surface and decreased with increasing depth (Figure 3 and Table 1). The resulting SOC changes were significantly different from 0 (95% CI did not cross 0) at all depths and were symmetrical but mirrored. The result is a large overall increase of SOC stocks down to 90 cm depth when converting cropland to grassland and losses when converting grassland to cropland (Figure 4).

**TABLE 1:** Summary statistics for Figure 3. Soil organic carbon stock change for each of the six land‐use change directions outlined in this study with bootstrapped 95% confidence intervals of the respective mean.

	Absolute SOC stock change following land‐use change (Mg C ha^−1^)					
	Cropland to grassland	Grassland to cropland	Cropland to forest	Forest to cropland	Grassland to forest	Forest to grassland
Litter			19.7 ± 0.4	−20.2 ± 1.1	20.6 ± 1.1	−19.8 ± 1.0
0–10 cm	21.5 ± 0.4	−21.2 ± 1.4	19.1 ± 0.4	−16.3 ± 0.8	−2.2 ± 1.5	4.3 ± 0.7
10–30 cm	10.9 ± 0.6	−8.9 ± 2.7	−5.2 ± 0.6	6.6 ± 0.9	−15.7 ± 3.6	14.4 ± 1.0
30–60 cm	7.7 ± 0.6	−6.0 ± 2.0	3.9 ± 0.7	−3.7 ± 0.9	−4.2 ± 3.4	4.1 ± 0.9
60–90 cm	4.5 ± 0.4	−4.6 ± 2.0	2.9 ± 0.5	−1.3 ± 0.7	−1.2 ± 2.4	2.2 ± 0.7
Overall	44.6 ± 1.1	−40.7 ± 4.6	40.4 ± 1.2	−34.9 ± 2.2	−2.7 ± 6.4	5.2 ± 2.2
Mineral soil	44.6 ± 1.1	−40.7 ± 4.6	20.7 ± 1.6	−14.7 ± 1.8	−23.3 ± 6.3	25.0 ± 1.9

**FIGURE 5:**
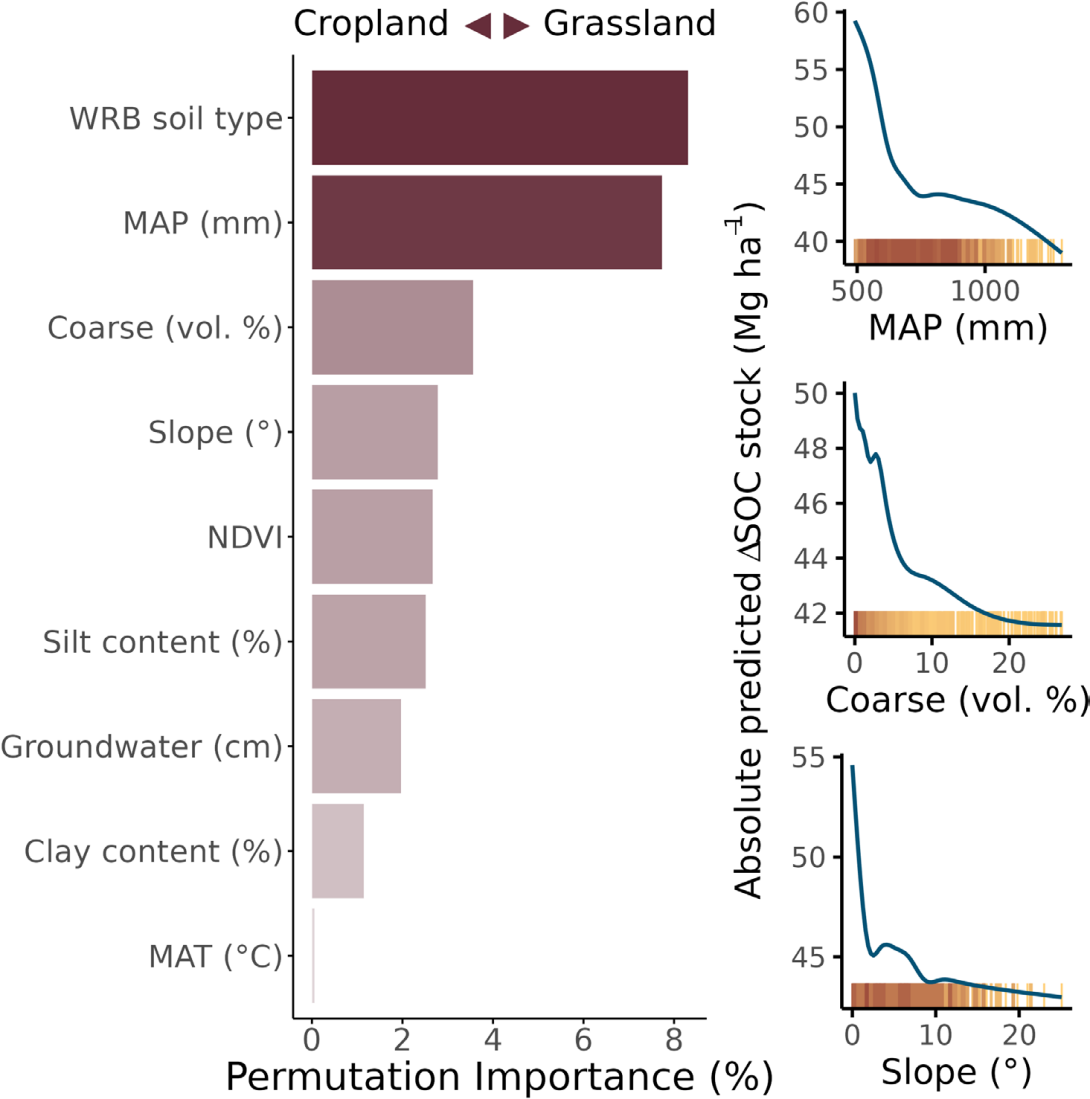
Drivers of site‐specific differences in the magnitude of soil organic carbon (SOC) change following land‐use change between cropland and grassland. How variation in values of the top three numerical variables affected predicted SOC stock change following LUC are shown on the right (accumulated local effect). Each yellow tick along the bottom of the ALE plots represents a site, with darker colors indicating higher density of sites.

Differences in reference soil groups explained the most variation in the magnitude of SOC stock change to 90 cm depth between sites following LUC between cropland and grassland and vice versa (Figure 5), with Gleysols and Fluvisols changing the most (absolute change), and Luvisols and Vertisols soils changing the least (Figure S18). After the reference soil group, mean annual precipitation and coarse content were the next most important drivers of differences in SOC stock change. Sites with less precipitation tended to have greater changes in SOC stocks; the same is true of sites with a smaller volume of coarse fragments.

### 3.2 Change Between Cropland and Forest

Similar to LUC between cropland and grassland, the magnitude of SOC change in the mineral soil was greatest near the surface and decreased with increasing depth. That being said, patterns of change for the 10–30 cm depth were reversed; SOC stock was predicted to be lost when converting cropland to forest and gained when converting forest to cropland (Figure 3 and Table 1). In both subsoil depths (30–60 cm and 60–90 cm), SOC stocks once again increased when converting cropland to forest and decreased when converting forest to cropland. Loss or gain of a litter layer accounts for more than half of the overall SOC stock change between cropland and forest and vice versa (Figure 4).

**FIGURE 6:**
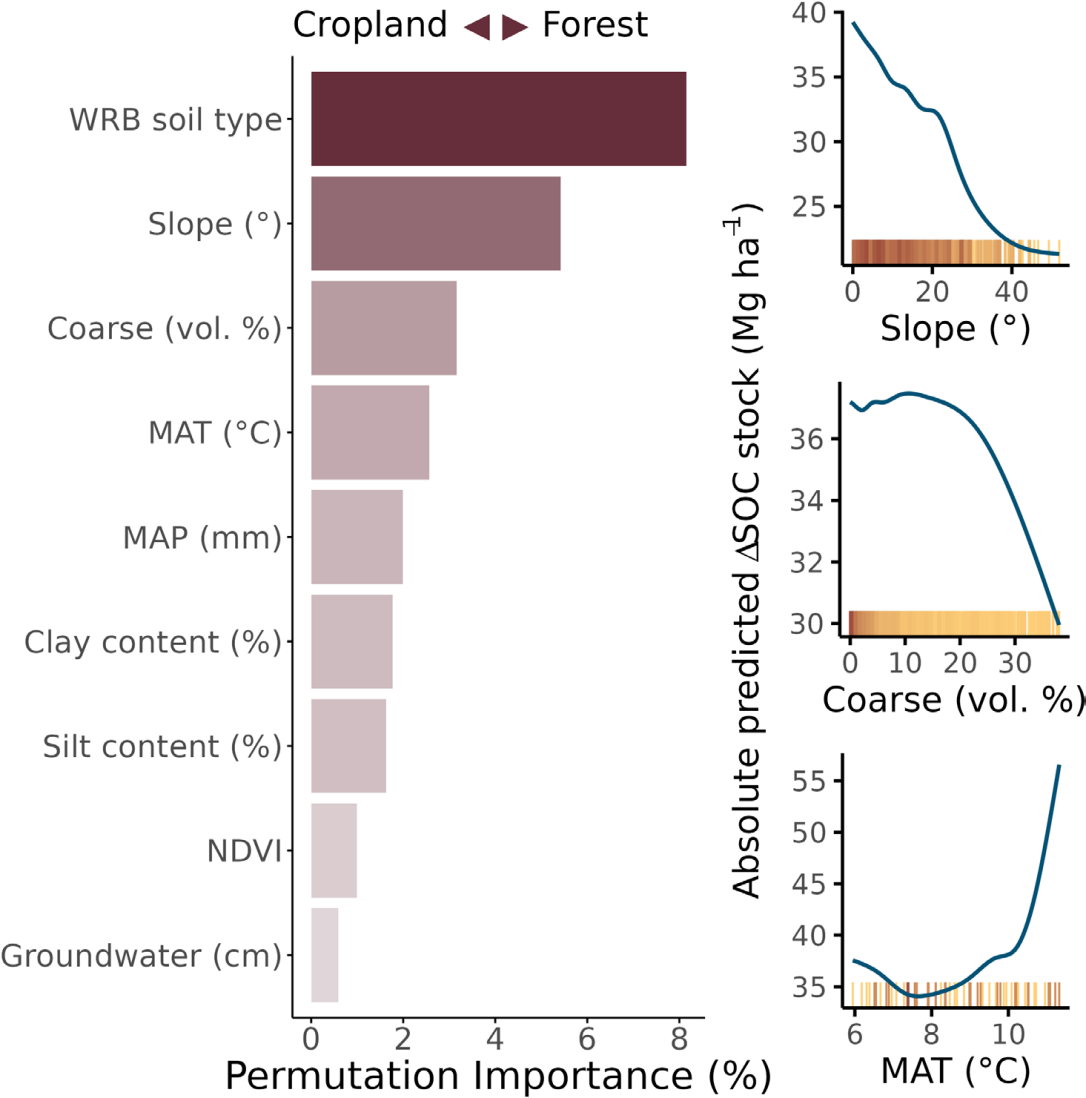
Drivers of site‐specific differences in the magnitude of soil organic carbon (SOC) change following land‐use change between cropland and forest How variation in values of the top three numerical variables affected predicted SOC stock change following LUC are shown on the right (accumulated local effect). Each yellow tick along the bottom of the ALE plots represents a site, with darker colors indicating higher density of sites.

Differences in reference soil group explain the most variation in the magnitude of SOC stock change to 90 cm depth between sites following LUC between grassland and forest and vice versa (Figure 6), with Gleysols and Fluvisols changing the most, and Chernozems and Phaeozems changing the least (Figure S19). The slope of a study site and the coarse fraction were the next most important factors. Flatter sites and sites with smaller volumes of coarse fragments tended to have greater changes in SOC stocks.

### 3.3 Change Between Grassland and Forest

While the majority of the SOC stock changes between grassland and forest took place in topsoil, the SOC change at the 0–10 cm depth was comparably small, with the majority of the change taking place at the 10–30 cm depth (Figure 3). Land‐use change effects on SOC stocks were significantly different from 0 for all depths and LUC directions except the 60–90 cm depth of grassland to forest LUC. Loss or gain of the litter layer offset most of the change in the mineral soil, such that the overall effect of grassland to forest conversion was not significantly different from 0 but is significant in the mineral soil alone (Figure 4).

**FIGURE 7:**
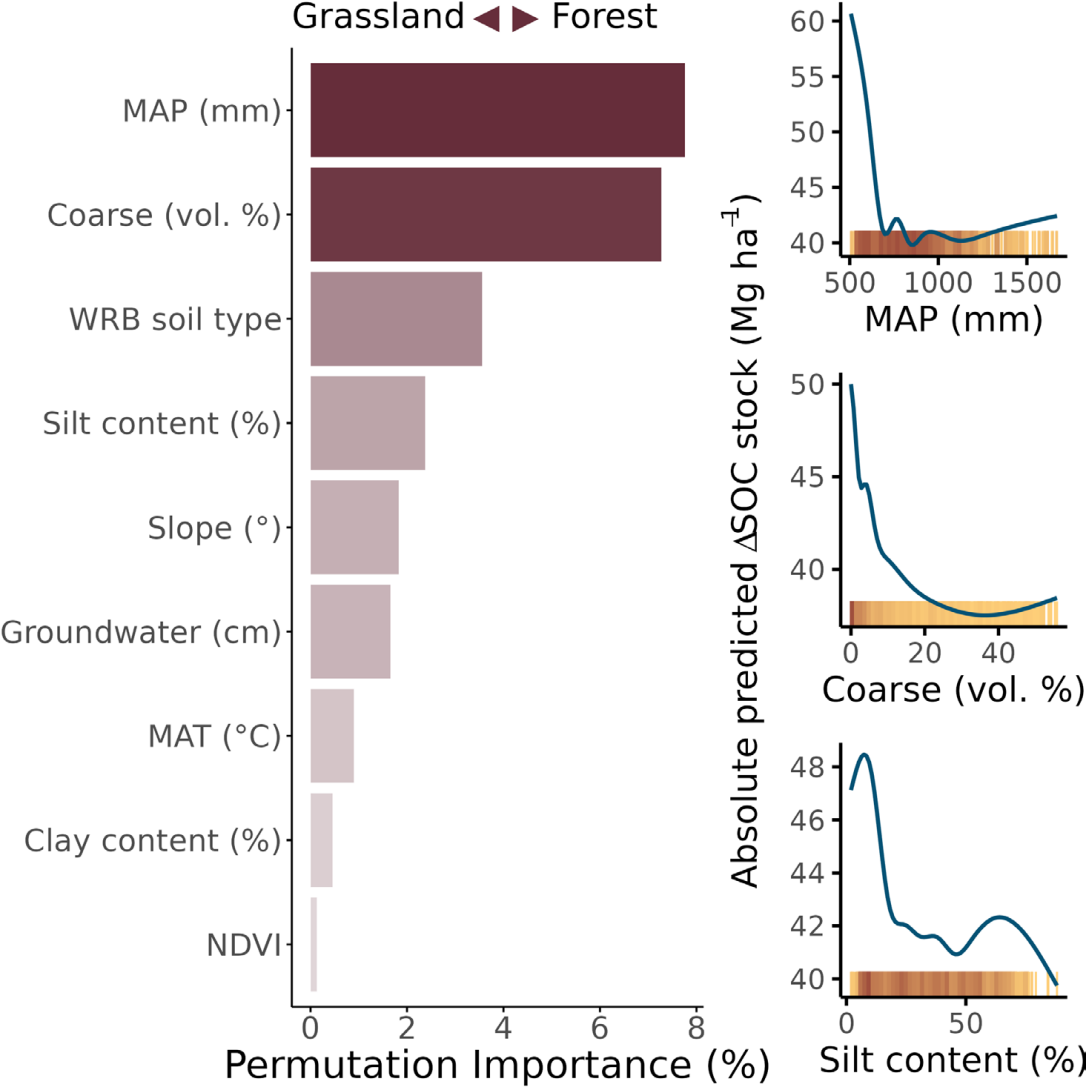
Drivers of site‐specific differences in the magnitude of soil organic carbon (SOC) change following land‐use change between forest and grassland. How variation in values of the top three numerical variables affected predicted SOC stock change following LUC are shown on the right (accumulated local effect) Each yellow tick along the bottom of the ALE plots represents a site, with darker colors indicating higher density of sites.

The coarse fraction explained the most variation in the magnitude of SOC stock change to 90 cm depth following LUC between grassland and forest and vice versa (Figure 7); sites with less coarse fraction tended to experience greater change. Mean annual precipitation and drought index were next most important, both with greatest change occurring at sites with smaller values. Reference soil group follows closely, however, with Anthrosols and Regosols changing the most, and Chernozems and Phaeozems changing the least (Figure S20).

### 3.4 Subsoil Contribution to Land‐Use Change Effects

The magnitude of soil organic carbon stock change in the subsoil compared to initial SOC stock differed by land‐use change type (Table 2). The proportion of change in the subsoil compared to the whole soil profile, however, was within confidence intervals of each other across all three LUC pairings. For LUC between cropland and grassland, subsoil SOC change accounted for 29% ± 4% of the overall change; SOC change in subsoils of LUC between cropland and forest accounted for 34% ± 4% of the overall change; and SOC change in the subsoils of LUC from grassland to forest and vice versa accounted for 25% ± 9% of the overall change. Finally, the average subsoil effect of LUC on SOC stock for LUC between cropland, grassland, and forest is 29.6% ± 3.5%.

**TABLE 2:** Soil organic carbon change relative to pre‐LUC SOC stocks for topsoil (0–30 cm) and subsoil (30–90 cm) for each of the six land‐use change directions outlined in this study with bootstrapped 95% confidence intervals of the respective mean.

	SOC stock change, relative to pre‐LUC SOC, following land‐use change (%)					
	Cropland to grassland	Grassland to cropland	Cropland to forest	Forest to cropland	Grassland to forest	Forest to grassland
Topsoil	58 ± 3	−34 ± 3	25 ± 2	−17 ± 2	−19 ± 4	29 ± 3
Subsoil	45 ± 5	−24 ± 6	25 ± 5	−18 ± 4	−12 ± 10	22 ± 6

## 4 Discussion

Converting land from one use to another has long‐lasting effects on SOC stocks and the soil physicochemical properties that mediate the cycling of C in the soil (Emde et al. 2024; Hobley et al. 2017; Schneider et al. 2021). Previous literature has often focused on tracking such changes only in the top 20 to 30 cm of soil because the topsoil reacts most strongly to changes in soil processes (Poeplau and Don 2013; Zhang et al. 2024). We have found that, while it is largely true that most change happens in the topsoil, a significant portion of LUC‐induced change may occur at soil depths below 30 cm (Figure 3). If subsoils are not considered when assessing the impact of LUC on SOC stocks, approximately one‐third of the SOC change is left unaccounted for. Depending on the scale of the LUC in question, this could account for millions of tonnes of unaccounted CO2 fluxes. For example, the largest share of LUC in Germany for 2021 was the conversion of 1.4 million ha of mineral cropland soils to grasslands (UNFCCC 2021). If we consider the average SOC stock change for cropland to grassland LUC for the two subsoil depths from this study (7.7 Mg C ha^−1^ for the 30–60 cm depth and 4.5 Mg C ha^−1^ for the 60–90 cm depth), this amounts to approximately 17 million tonnes of potentially unaccounted for CO_2_ sink due to SOC changes following LUC as new grasslands build up SOC.

Beyond examining SOC changes due to LUC in the subsoil, the site‐specificity of the modeling approach used in this research provides a unique opportunity to link SOC change with site‐specific drivers of that change. To that end, we found that the WRB soil group was the most important site characteristic for determining the magnitude of SOC change for LUC across all LUC directions. For LUC including cropland, Gleysols and Fluvisols had the greatest changes following LUC. These are soils that are significantly influenced by water and tend to be SOC rich. Where Gleysols are formed in waterlogged conditions with poor drainage, Fluvisols develop in alluvial environments like floodplains, where periodic waterlogging is common. For LUC between grassland and forest, Anthrosols and Regosols showed the largest changes. Both soils typically have limited development and have human influence. Anthrosols, in fact, are diagnostically human impacted, but may retain some characteristics of their parent material. Regosols, meanwhile, are particularly young soils and tend to occur in human impacted areas (International Union of Soil Sciences IUSS 2022).

### 4.1 Land‐Use Change Between Cropland and Grassland

Our results show that when sites with annual cropping systems are converted to grassland, they gain an average of 44.6 ± 1.1 Mg C ha^−1^ if given enough time to reach equilibrium state. A similar SOC stock change (−40.7 ± 4.6 Mg C ha^−1^) occurs when converting meadows and pastures to annual croplands (Figure 4 and Table 1). The greatest portion of this change occurs at the surface and decreases with increasing depth, with significant changes at all depths down to the sampled depth of 90 cm (Figure 3 and Table 1).

The 40.7 Mg C ha^−1^ overall loss (22.0% ± 4.0% relative loss) of SOC stocks when converting grasslands to croplands in this study is in line with Li et al. (2018), one of the few studies from the literature that examined similar soils and similar depth. Li et al. (2018) found an approximately 26% loss to 1 m depth for European sites from a global meta‐analysis. Poeplau and Don (2013), meanwhile, found no significant change in SOC stocks in the subsoil (30–80 cm) of European grassland to cropland conversion. While studies extending past 20 or 30 cm depth are extremely limited, reports of topsoil SOC change are plentiful and range from approximately 10% losses in tropical climates (Don et al. 2011) to more than 30% losses in temperate climates (Huang et al. 2024; Liang et al. 2023; Poeplau et al. 2011). This study shows approximate losses of 32% ± 5% in the topsoil and 23% ± 8% in the subsoil (Table 2). For LUC in the opposite direction, from cropland to grassland, Li et al. (2018) reported an average SOC stock increase of 37% to 1 m in European soils, approximately half of the 72% ± 3% increase found in this study. Poeplau and Don (2013), once again, found no significant increase in subsoil (30–80 cm) SOC following cropland to grassland LUC in Europe. Topsoils SOC change was, once again, variable; ranging from increases of about 35% to more than 100% (Don et al. 2011; Huang et al. 2024; Poeplau et al. 2011). This study shows approximate SOC stock increases of 60% ± 2% for topsoil and 45% ± 4% in the subsoil.

Differences in the reported SOC change following LUC from cropland to grassland and vice versa may be partially attributable to variations in the time since LUC (site age) and equilibrium status of the pre‐ and post‐LUC sites. In order to assess the full magnitude of the effect of LUC on SOC stocks, sites must be at SOC equilibrium with regard to LUC prior to and after LUC. Without accounting for equilibrium status, it is possible that the sites being compared are still losing or gaining C as a result of historic LUC (Emde et al. 2024). With studies increasingly reporting LUC effects for many decades following LUC, studies that report SOC change for younger sites will report smaller LUC‐induced SOC change than those including older sites. With the difficulty of obtaining such long‐term land‐use histories, it is entirely possible that much of the variation in reported LUC effects in the current literature is due to unconfirmed equilibrium status.

The depth dynamic of SOC change following LUC is well established, and the patterns shown in this study mirror the consensus reflected in the current literature. The greatest absolute changes occur where there is the most SOC; that is, closest to the soil surface where the plant roots responsible for drawing C into the soil are most abundant (Jacobs et al. 2020; Poeplau et al. 2021). Both croplands and grasslands are intensively managed in Germany, but perennial grasses have persistent foliage and dense, deep root systems with relatively high root turnover, while annual crops have root systems that input less C to the soil and biomass that is largely removed with harvest (Goss and Watson 2003; Warren Raffa et al. 2015). Tillage and other mechanical disruption in croplands may play a role to some degree in C losses following conversion of grassland to cropland, but recent publications more often point to SOC formation efficiency (i.e., organic matter quality) as the key difference between C gained from crop residues and grasses (Jacobs et al. 2020; Poeplau et al. 2021).

Once C has entered the soil, its subsistence is mediated by a number of factors, many of which are related to the ability of a soil to form aggregates (Even and Cotrufo 2024). Soil aggregates not only physically protect enclosed organic matter from mineralization, but also enhance soil properties responsible for both crop productivity and carbon cycling (Goebel et al. 2009; Horn et al. 1994). Our study showed that the WRB reference soil group drove the site‐specific differences in magnitude of change following LUC from cropland to grassland and vice versa, with the largest absolute changes occurring in Gleysols and Fluvisols. Both Gleysols and Fluvisols tend to reside in lowland areas and floodplains that are inherently rich in organic matter, have groundwater levels closer to the surface, and show enhanced ability to produce aggregates (Monreal et al. 1997; Radziuk and Świtoniak 2022).

Periodic waterlogging limits oxygen availability for decomposers, thereby increasing mean residence time of SOC due to reduced activity. When drained for cropland use, microbial activity is no longer inhibited by anoxic conditions and the resultant decrease in mean residence time leads to a loss of SOC (Argiroff et al. 2017). Furthermore, wet‐dry cycles combined with the presence of soil colloids and the specific mineralogy of Gleysols and Fluvisols enhance aggregate formation and stability (Tang et al. 2024; Wasner et al. 2024). Disrupting aggregates, as with the introduction of conventional agricultural management and tillage, stands to enhance carbon loss by diminishing the positive effects of aggregation on the soil (Six et al. 2000; Ye et al. 2020). The opposite is true when changing croplands to grassland (Xiao et al. 2021). The impact of reference soil group is thereby twofold: first, sites with higher SOC stocks (potentially due to the influence of groundwater) tend to experience larger absolute changes following LUC by merit of having more C to shift and second, through changes in soil aggregation.

Because German agriculture is largely unirrigated, mean annual precipitation (MAP) plays an integral role in plant productivity by determining when and to what degree crops and grasses receive water (Destatis 2019). As such, precipitation is also related to NDVI, an indicator of vegetation growth and density (Du et al. 2017). Coarse fragment content, meanwhile, is a key component in soil porosity and, therefore, hydrological properties and root infiltration (Lai et al. 2021; Sekucia et al. 2020). Our results show that, on average, sites with less precipitation, lower NDVI, and lower coarse fragment content had larger predicted changes of SOC stock following LUC (Figure 5). This suggests that limits placed on plant productivity are important drivers of SOC change following LUC.

### 4.2 Land‐Use Change to and From Forest

The overall magnitude of SOC change for LUC involving forests is largely driven by the addition or removal of a litter layer. Converting cropland sites to forest increases SOC overall (39 ± 2 Mg C ha^−1^), but more than half of that additional SOC in forests is retained in the litter layer. Converting forests to cropland, meanwhile, results in a loss of 33 ± 2 Mg C ha^−1^, the majority of which stems from the loss of a litter layer. With grasslands, the impact of the litter layer on overall SOC change is even more pronounced, such that including the litter layer all but compensates for the SOC change in the mineral soil (Figure 3 and Table 1).

#### 4.2.1 Change Dynamics for LUC Between Cropland and Forest

The litter layer is unique to forest ecosystems and acts as an extension of the surface layer of the mineral soils (Chakravarty et al. 2022; Neumann et al. 2018; Walkiewicz et al. 2021). While it is generally understood that, depending on soil types and tree species composition, leaf litter contributes about as much to SOC as belowground inputs in forests (Beidler et al. 2023; Crow et al. 2009; Rasse et al. 2005), previous LUC studies on SOC in forest soils often either omit it or do not note whether the litter layer is included in the analysis. Roughly speaking, we observed an inverse relationship between the SOC stock of the litter layer and of the mineral soil at 0–10 cm depth in forests across Germany (Grüneberg et al. 2019). Where litter layer SOC stocks were high, 0–10 cm SOC stocks were low, and vice versa.

The current literature reports that mineral topsoil SOC stocks change by approximately one‐third of their pre‐LUC stock when converting land between cropland and forest in temperate regions (Huang et al. 2024; Liang et al. 2023). Poeplau and Don (2013) noted 22 ± 11 Mg ha^−1^ losses of litter layer C when clearing European forests for cropland use, and no significant loss of SOC below 30 cm depth. Our results show litter losses in line with Poeplau and Don (2013) (20.2 ± 1.1 Mg C ha^−1^) but smaller mineral soil C changes in the topsoil (closer to 20% change). Finally, we found significant subsoil losses of C when converting forest to cropland (18% ± 4%) and increases when converting cropland to forest (25% ± 5%; Table 2).

Assuming that deforestation is followed by common national cropland management practices, the soil would be plowed, mixing the upper 30 cm of soil and homogenizing the SOC content in the plowed layer. As a consequence, converted forest sites would lose large amounts of SOC at the 0–10 cm depth but gain SOC for the following 10–30 cm depth. Although croplands tend to have short‐lived crops with root systems that prioritize crop biomass and have inhomogeneous soil cover, forests have enormous amounts of aboveground woody biomass, consistent foliar litter groundcover, and deep, but patchy, root systems (Kochiieru et al. 2023; Pinno and Wilson 2013). Foliar litter, particularly the needles of coniferous trees, has a higher C:N ratio (i.e., poorer quality; Eremija et al. 2024) and high tannin content that can acidify soils during pedogenesis (Berg and Matzner 1997; Grüneberg et al. 2014, 2019; Meiwes et al. 1986). These acid soils containing OM from the litter layer with low N availability inhibit microbial activity and can lead to leaching of soil nutrients and metal cations associated with mineral stabilization of C (Berggren et al. 1998; Kindler et al. 2011; Neumann et al. 2018; Ulrich 1986). In croplands, soil pH is often higher and C inputs to the soil are mainly in the form of comparably sparse crop roots (narrower C:N ratio, less C input), conditions that favor C loss (Kätterer et al. 2011; Xu et al. 2023). As such, when forests are converted to cropland, large amounts of relatively undecomposed organic matter become vulnerable to mineralization.

Reference soil group was identified as the main driver behind site‐specific changes in the magnitude of SOC change between cropland and forest, with the largest changes taking place in Gleysols and Fluvisols (Figures 6 and S8). While the smallest absolute changes took place in Chernozems and Phaeozems, the forest area under Chernozems is notably much smaller than under cropland (there are only 14 chernozemic forest sites). Many C storage dynamics rely, in one form or another, on a soil's capacity to form aggregates. These dynamics and how they relate to LUC involving cropland are discussed in full above (Section 4.1). In brief, Gleysols in particular have an enhanced capacity for stable aggregate formation, and both Gleysols and Fluvisols tend to lie in lowland areas prone to periodic waterlogging (Monreal et al. 1997; Radziuk and Świtoniak 2022). When such forests are drained for cropland use, the enhanced activity of microbial decomposers and leaching of dissolved organic carbon lead to the loss of SOC (Paul et al. 2022; Yang, Drury, et al. 2022). Additionally, disruption of stable aggregates on sites previously under forest land use and the fact that these soils are likely to be higher in SOC because of where they reside in the landscape likely accounts for the larger‐than‐average changes in Gleysols and Fluvisols compared to other reference soil groups (Gajić et al. 2010). As forests establish on sites that were previously cropland, natural processes re‐establish soil structure and enhance SOC stocks.

#### 4.2.2 Change Dynamics for LUC Between Grassland and Forest

Forest LUC involving grasslands offers a diverse comparison. Huang et al. (2024) found that topsoil SOC stocks increased by 22% (16% to 28%) for grassland to forest LUC in temperate zones globally and found no significant effect of LUC from forest to grassland. One of the few studies we found that included forest litter, Poeplau et al. (2011), estimated that converting grassland to forest resulted in a similarly large SOC stock gain to about 40 cm depth (28% ± 11%), all of which was accounted for by the addition of a litter layer. In fact, without the litter layer, they found a 6.5% ± 23% decrease. Our results, meanwhile, show 19% ± 4% losses in the mineral topsoil for grassland to forest LUC (3% ± 7% increase when including the litter layer) and 29% ± 3% increases for forest to grassland LUC (5% ± 7% increase when including the litter layer). It is likely that the diversity of LUC effect estimates in LUC including forests is at least partially due to the diversity in forest landscapes and ecosystems themselves (Darro et al. 2022; Liu et al. 2024; Yang, Diao, et al. 2022) but may also be impacted by the equilibrium status of the croplands and grasslands before and after LUC as discussed in Section 4.1.

While forest C inputs are split between aboveground deposition by litterfall and belowground root dynamics, grassland inputs are predominantly derived from a comparably high root turnover rate and rhizodeposition of dense root systems (Bowden et al. 2024; Gill and Jackson 2000; Yang et al. 2023; Y. Zhang et al. 2023). Since forests and grasslands both have high SOC stocks near the surface, the SOC change due to LUC between grassland and forest is small for the 0–10 cm depth increment. Below 10 cm, however, greater belowground SOC input to grassland soils, coupled with the potential leaching of SOC from forest soils, reduces SOC in forest soils relative to grasslands following LUC.

Although German grasslands are managed systems, they are likely less frequently drained and not subject to the same disturbances due to tillage as croplands (Czerwiński et al. 2015). As such, natural processes that enhance soil structure, like aggregation, remain relatively undisturbed. Instead, the coarse content and annual precipitation play a dominant role in determining how SOC change following LUC differs between sites for LUC from grassland to forest and vice versa (Figure 7). In our dataset, forest soils had higher average coarse content than grasslands (6.8% volume vs. 1.4% for grassland). In addition to potential differences in hydrological properties and increased soil porosity (discussed in Section 4.1), since SOC can only exist in the fine soil, rocky soils (i.e., higher coarse content) typically have lower SOC stocks. Meanwhile, grassland productivity relies heavily on precipitation. Where water from precipitation is readily available, aboveground biomass is increased (Q. Guo et al. 2012), but when precipitation is limited, grasses invest more heavily in root growth to seek out available water (Alon and Sternberg 2019; Eziz et al. 2017; Guasconi et al. 2023). It follows that grasslands under lower mean annual precipitation may have more extensive root systems and, therefore, higher SOC (Guasconi et al. 2023).

### 4.3 Strengths and Limitations

With data‐driven reciprocal modeling, we are able to predict the site‐specific SOC change at equilibrium for sites undergoing LUC based on site properties alone. To measure SOC changes on the same plot from equilibrium to equilibrium otherwise would take many decades to more than a century (Emde et al. 2024; Li et al. 2018; Springob et al. 2001). As such, data‐driven reciprocal modeling has the potential to be an incredibly powerful tool in the SOC accounting toolkit. Moreover, because it can estimate SOC change on a per‐site basis.

While using machine learning modeling techniques to determine SOC change following LUC allows us to examine scenarios for which experimentation is unfeasible, it also complicates interpretation and introduces potential for extrapolation (i.e., predicting outside the range of known data) and bias. The data‐driven reciprocal modeling approach used here attempts to account for difficulties in interpretation by producing a second, post hoc, model that assesses the potential drivers of SOC change. Bias, on the other hand, is at least partially addressed in this study by using a stacked ensemble modeling approach rather than a single model type (Wolpert 1992). However, there is still the potential, albeit reduced, for regression to the mean or “shrinkage”; a problem typical of tree‐based machine learning models, including those in the ensemble. If present, shrinkage could result in overestimation of SOC change following LUC at the lower end of measured SOC stock values, and underestimation at the upper end of measured SOC stock values. Additionally, to prevent extrapolation, we used a data‐driven, area of applicability technique to systematically exclude sites that are too different from the training sites to be predicted without extrapolation. This approach ensures that we only compare like with like, but it also selects for levels of site properties common to the target group.

Most of the current literature shows similar or smaller LUC effects than we have reported here, particularly when considering the subsoil. This may partially be a product of limiting extrapolation in the modeling pipeline, as described above, but perhaps more so a limitation of available long‐term data. Because soil organic carbon continues to change for many decades to more than a century (Emde et al. 2024; Li et al. 2018; Springob et al. 2001), studies that look at LUC where sites underwent LUC even a few decades ago may only be taking a snapshot of the SOC change along the way to a new equilibrium state. Here, because we included only sites at equilibrium with regard to SOC according to extensive historical land‐use records, we are confident that we have captured the full, larger effect of LUC on SOC.

### 4.4 Conclusions

With approximately 30% of the LUC effect on SOC stocks occurring in the subsoils, it is clear that accounting for only topsoil excludes a large portion of the C change occurring in agricultural and forest soils. Furthermore, the current literature more often than not reports SOC change following LUC for a few decades at most; a time period insufficient to reach new SOC equilibrium. When taken together, this leads to the conclusion that the impact of LUC on SOC has likely been considerably underestimated. Determining how much soil C is lost as a result of LUC from more natural systems to cropland or gained following implementation of measures to enhance soil C storage relies on proper accounting of the full magnitude of C change. As such, this potential underestimation has far‐reaching implications for carbon accounting measures and the emissions reporting schemes upon which environmental and agricultural policy decisions rely.

## Author Contributions

**David Emde:** conceptualization, data curation, formal analysis, methodology, visualization, writing – original draft. **Ali Sakhaee:** methodology, writing – review and editing. **Christopher Poeplau:** conceptualization, writing – review and editing. **Axel Don:** conceptualization, writing – review and editing. **Marc Scherstjanoi:** data curation, writing – review and editing. **Nicole Wellbrock:** data curation, writing – review and editing. **Florian Schneider:** conceptualization, data curation, methodology, supervision, writing – review and editing.

## Conflicts of Interest

The authors declare no conflicts of interest.

## Supporting information


**Table S1:** Variables used in each model with sources.
**Figure S1:** Distribution of numeric variables by land‐use type for the 0–10 cm depth increment.
**Figure S2:** Distribution of numeric variables by land‐use type for the 10–30 cm depth increment.
**Figure S3:** Distribution of numeric variables by land‐use type for the 30–60 cm depth increment.
**Figure S4:** Distribution of numeric variables by land‐use type for the 60–90 cm depth increment.
**Figure S5:** Distribution of numeric variables by land‐use type for the litter layer.
**Table S2:** Performance metrics for all SOC models produced during Step 2 of the data‐driven reciprocal modeling pipeline.
**Figure S6:** Model confidence via interquartile range of model output for the ensemble model predicting SOC stock for cropland to grassland land‐use change.
**Figure S7:** Model confidence via interquartile range of model output for the ensemble model predicting SOC stock for grassland to cropland land‐use change.
**Figure S8:** Model confidence via interquartile range of model output for the ensemble model predicting SOC stock for cropland to forest land‐use change.
**Figure S9:** Model confidence via interquartile range of model output for the ensemble model predicting SOC stock for forest to cropland land‐use change.
**Figure S10:** Model confidence via interquartile range of model output for the ensemble model predicting SOC stock for grassland to forest land‐use change.
**Figure S11:** Model confidence via interquartile range of model output for the ensemble model predicting SOC stock for forest to grassland land‐use change.
**Figure S12:** Variable importance for SOC stock models for cropland to grassland land‐use change (Step 2).
**Figure S13:** Variable importance for SOC stock models for grassland to cropland land‐use change (Step 2).
**Figure S14:** Variable importance for SOC stock models for cropland to forest land‐use change (Step 2).
**Figure S15:** Variable importance for SOC stock models for forest to cropland land‐use change (Step 2).
**Figure S16:** Variable importance for SOC stock models for grassland to forest land‐use change (Step 2).
**Figure S17:** Variable importance for SOC stock models for forest to grassland land‐use change (Step 2).
**Figure S18:** ALE for WRB reference soil group for crop<>grass effect size.
**Figure S19:** ALE for WRB reference soil group for crop<>forest effect size.
**Figure S20:** ALE for WRB reference soil group for grass<>forest effect size.

## Data Availability

The R code and dataset used in this manuscript are available on GitHub (https://github.com/dsemde/drivers‐SOC‐LUC) and Zenodo (https://doi.org/10.5281/zenodo.15225982).
