# Supplementary material for: Site‐Specific Drivers of Land‐Use Change Effects on Organic Carbon in German Agriculture and Forest Soils
Source: Glob Chang Biol. 2025 Oct 28;31(10):e70576. doi: 10.1111/gcb.70576 (PMC12559880; doi:10.1111/gcb.70576)

**Table S1**: Variables used in each model with sources.

| Model | Included variables | Source |
| --- | --- | --- |
| Litter layer | WRB reference soil group,  Mean annual temperature  Mean annual precipitation Drought index Elevation Slope | German ^1^ agricultural and ^2^ forest soil inventories  ^3^ German Meteorological Service ^3^ German Meteorological Service  ^3^ German Meteorological Service  ^4^ EcoDataCube  ^4^ EcoDataCube |
| Topsoil  (0-10cm, 10-30cm) | WRB reference soil group  Mean annual temperature  Mean annual precipitation  Drought index  Elevation  Slope  Groundwater level  Clay content Silt content  Volume of coarse fraction > 2 mm  Total inorganic carbon  Horizon parent material composition  For non-forest LUC also:  Soil pH in H2O  C:N ratio | German ^1^ agricultural and ^2^ forest soil inventories  ^3^ German Meteorological Service ^3^ German Meteorological Service  ^3^ German Meteorological Service  ^4^ EcoDataCube  ^4^ EcoDataCube  German ^1^ agricultural and ^2^ forest soil inventories  German ^1^ agricultural and ^2^ forest soil inventories  German ^1^ agricultural and ^2^ forest soil inventories  German ^1^ agricultural and ^2^ forest soil inventories  German ^1^ agricultural and ^2^ forest soil inventories  German ^1^ agricultural and ^2^ forest soil inventories  German ^1^ agricultural and ^2^ forest soil inventories  German ^1^ agricultural and ^2^ forest soil inventories |
| Subsoil (30-60cm, 60-90cm) | WRB reference soil group  Mean annual temperature  Mean annual precipitation  Drought index  Elevation  Slope  Groundwater level  Clay content  Silt content  Volume of coarse fraction > 2 mm  Total inorganic carbon  Horizon parent material composition  Soil horizon symbols  For non-forest LUC also:  Soil pH in H2O  C:N ratio | German ^1^ agricultural and ^2^ forest soil inventories  ^3^ German Meteorological Service  ^3^ German Meteorological Service  ^3^ German Meteorological Service  ^4^ EcoDataCube  ^4^ EcoDataCube  German ^1^ agricultural and ^2^ forest soil inventories  German ^1^ agricultural and ^2^ forest soil inventories  German ^1^ agricultural and ^2^ forest soil inventories  German ^1^ agricultural and ^2^ forest soil inventories  German ^1^ agricultural and ^2^ forest soil inventories  German ^1^ agricultural and ^2^ forest soil inventories  German ^1^ agricultural and ^2^ forest soil inventories  German ^1^ agricultural and ^2^ forest soil inventories  German ^1^ agricultural and ^2^ forest soil inventories |
| Post-hoc model | WRB reference soil group  Mean annual temperature  Mean annual precipitation  Drought index  Slope  Groundwater level  NDVI  Clay content  Silt content  Volume of coarse fraction > 2 mm | German ^1^ agricultural and ^2^ forest soil inventories  ^3^ German Meteorological Service  ^3^ German Meteorological Service  ^3^ German Meteorological Service  ^4^ EcoDataCube  German ^1^ agricultural and ^2^ forest soil inventories  ^4^ EcoDataCube  German ^1^ agricultural and ^2^ forest soil inventories  German ^1^ agricultural and ^2^ forest soil inventories  German ^1^ agricultural and ^2^ forest soil inventories |

**^1^** Poeplau, C., Don, A., Flessa, H., Heidkamp, A., Jacobs, A., & Prietz, R. (2020). Erste Bodenzustandserhebung Landwirtschaft – Kerndatensatz. https://doi.org/10.3220/DATA20200203151139

^2^ Wellbrock, N., Aydin, C.-T., Block, J., Bussian, B., Deckert, M., Diekmann, O., Evers, J., Fetzer, K. D., Gauer, J., Gehrmann, J., Kölling, C., König, N., Liesebach, M., Martin, J., Meiwes, K. J., Milbert, G., Raben, G., Riek, W., Schäffer, W., & Wolff, B. (2006). Bodenzustandserhebung im Wald (BZE II), Arbeitsanleitung für die Außenaufnahmen. https://www.openagrar.de/receive/timport_mods_00003021

^3^ Deutscher Wetterdienst. (2022, March 13). Open Data Server of the German Meteorological Service. German Meteorological Service. <https://opendata.dwd.de/> (1 km resolution)
^4^ Witjes, M., Parente, L., Križan, J., Hengl, T., & Antonić, L. (2023). Ecodatacube.eu: analysis-ready open environmental data cube for Europe. PeerJ, 11, e15478. (30 m resolution)


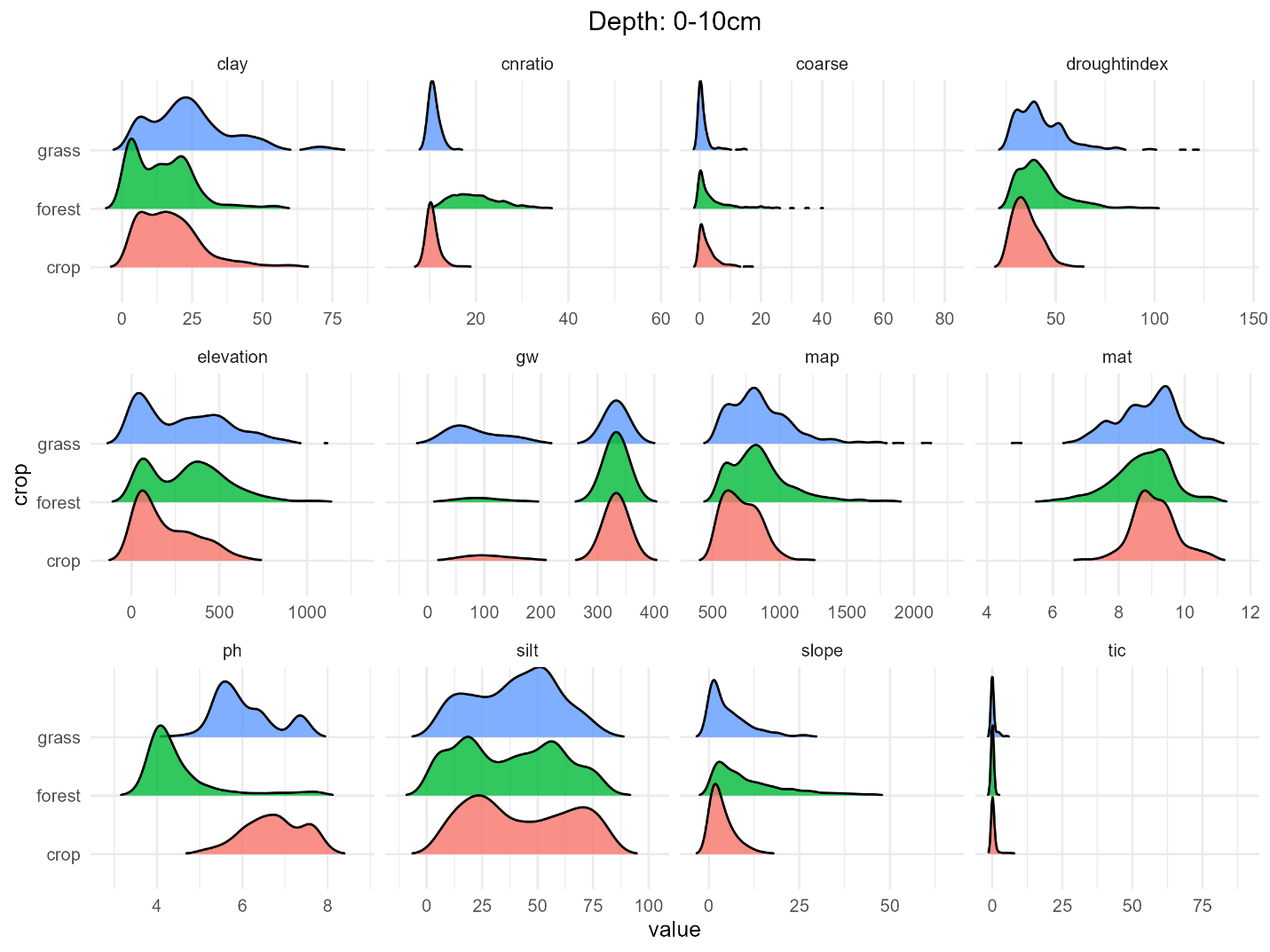


**Figure S1**: Distribution of numeric variables by land-use type for the 0-10 cm depth increment.


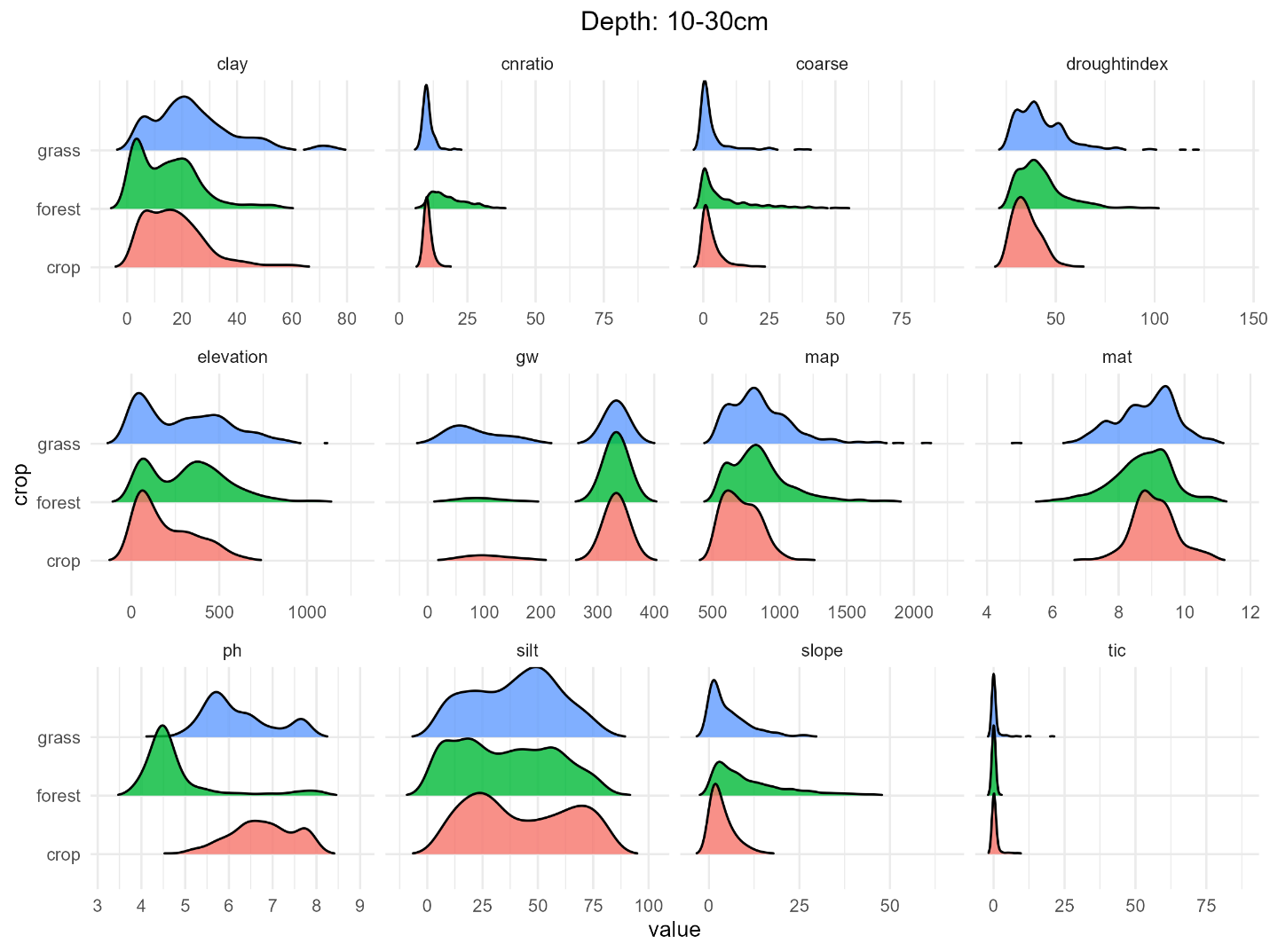


**Figure S2**: Distribution of numeric variables by land-use type for the 10-30 cm depth increment.


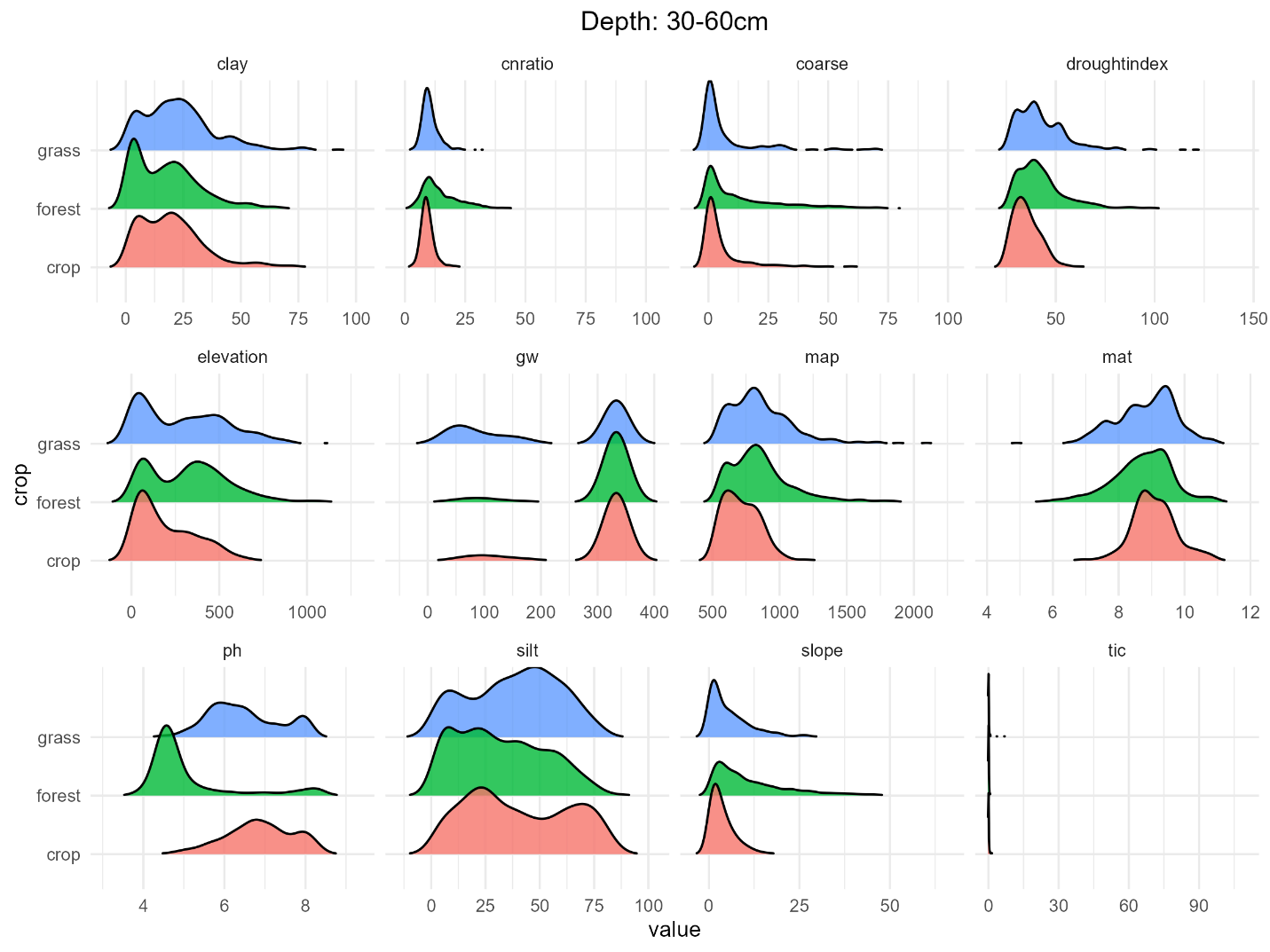


**Figure S3**: Distribution of numeric variables by land-use type for the 30-60 cm depth increment.


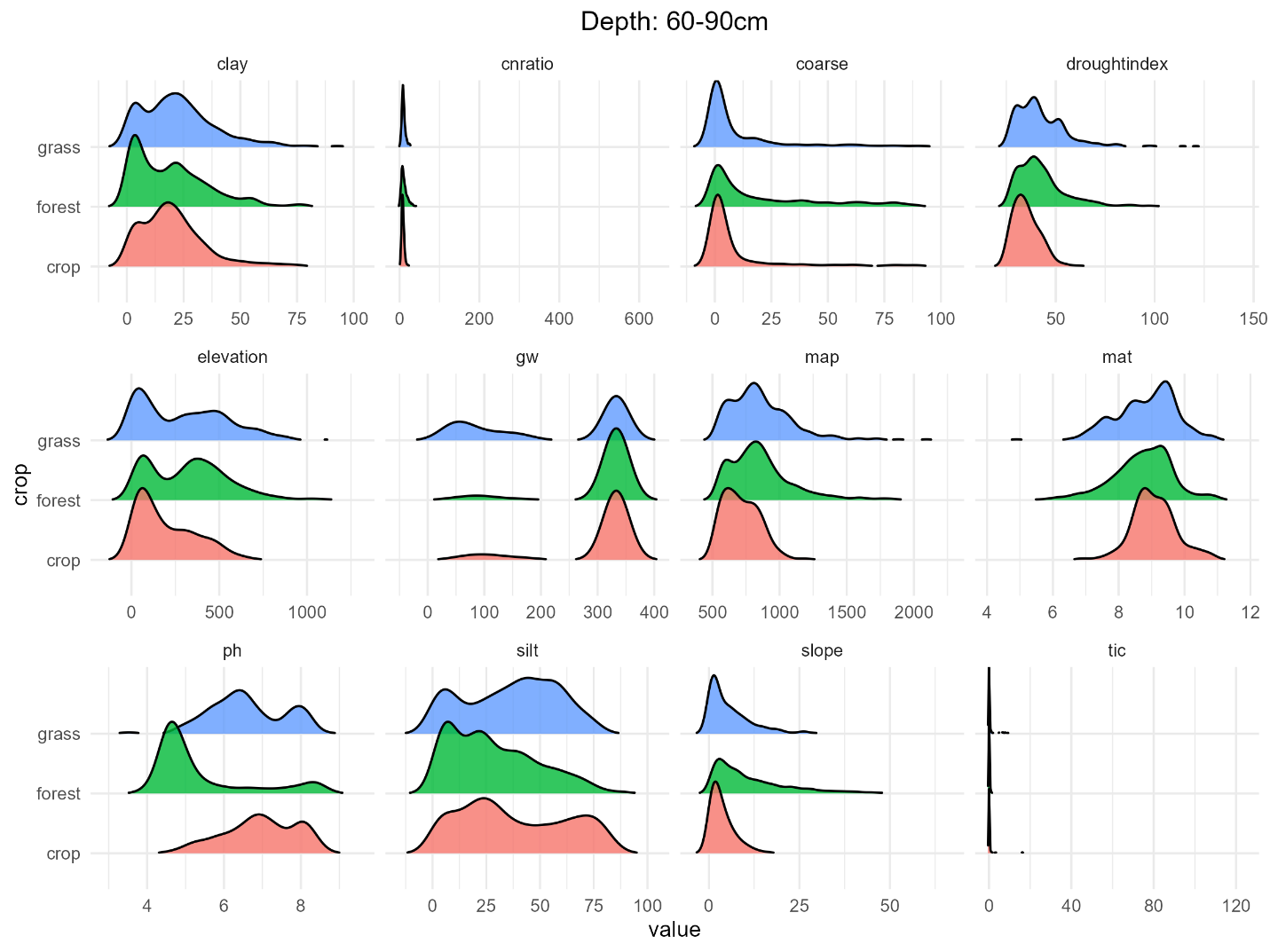


**Figure S4**: Distribution of numeric variables by land-use type for the 60-90 cm depth increment.


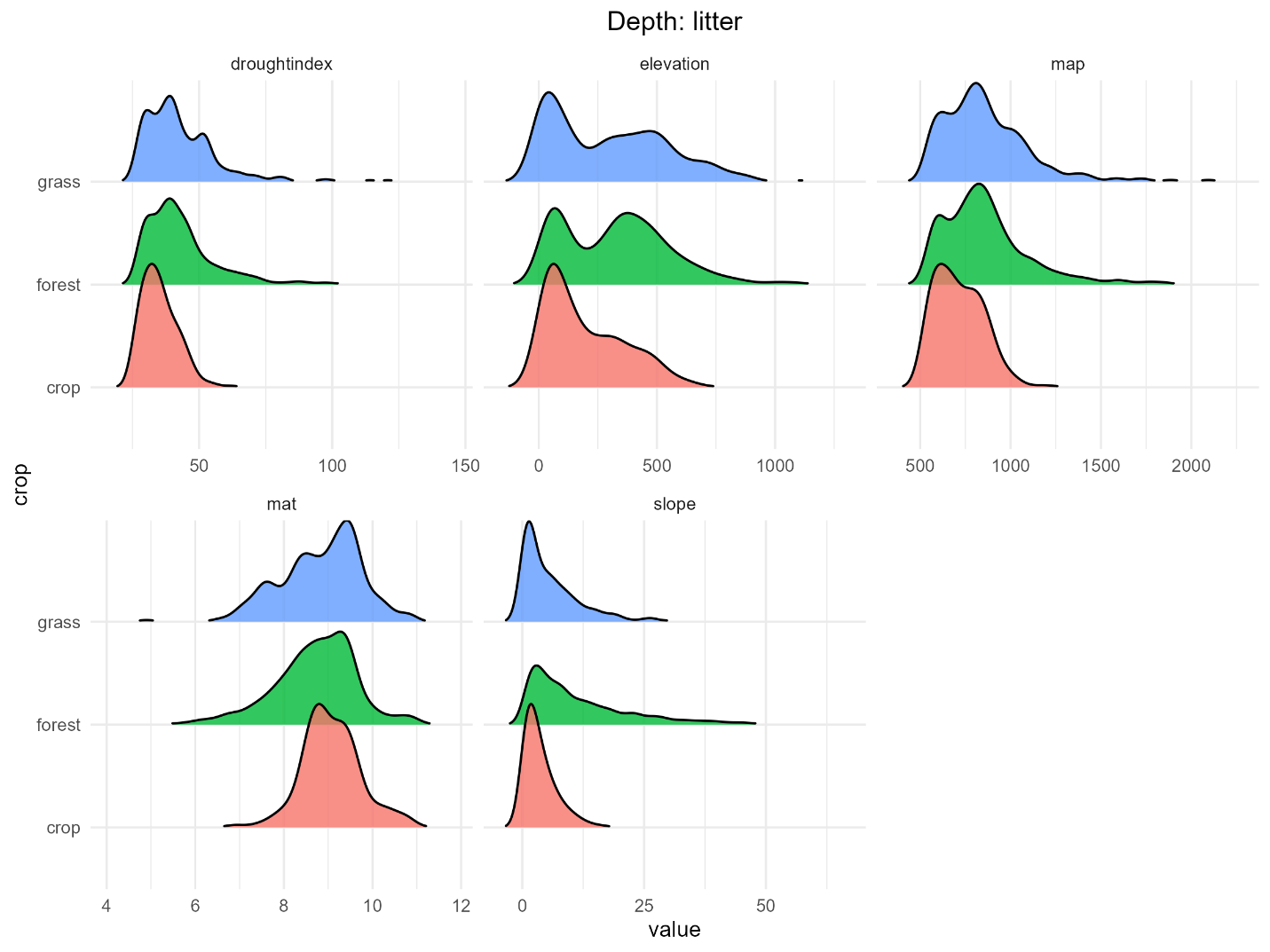


**Figure S5**: Distribution of numeric variables by land-use type for the litter layer.

**Table S2**: Performance metrics for all SOC models produced during Step 2 of the data-driven reciprocal modelling pipeline.

| Model | Depth (cm) | MAE | RMSE |
| --- | --- | --- | --- |
| Crop to grass | 0-10 | 7.3 | 9.3 |
|  | 10-30 | 14.5 | 19.4 |
|  | 30-60 | 12.5 | 20.4 |
|  | 60-90 | 8.9 | 14.8 |
| Grass to crop | 0-10 | 3.1 | 4.3 |
|  | 10-30 | 5.8 | 8.1 |
|  | 30-60 | 5.3 | 8.5 |
|  | 60-90 | 3.0 | 5.7 |
| Crop to forest | 0-10 | 8.6 | 11.3 |
|  | 10-30 | 9.5 | 13.7 |
|  | 30-60 | 8.1 | 14.5 |
|  | 60-90 | 5.3 | 13.2 |
|  | litter | 12.3 | 17.2 |
| Forest to crop | 0-10 | 3.46 | 4.94 |
|  | 10-30 | 6.58 | 9.52 |
|  | 30-60 | 5.97 | 9.62 |
|  | 60-90 | 3.41 | 6.58 |
| Grass to forest | 0-10 | 8.6 | 11.3 |
|  | 10-30 | 9.5 | 13.7 |
|  | 30-60 | 8.1 | 14.5 |
|  | 60-90 | 5.3 | 13.2 |
|  | litter | 12.3 | 17.2 |
| Forest to grass | 0-10 | 7.3 | 9.4 |
|  | 10-30 | 15.7 | 21.3 |
|  | 30-60 | 13.3 | 21.9 |
|  | 60-90 | 9.6 | 15.8 |

**Figure S6**: Model confidence via interquartile range of model output for the ensemble model predicting SOC stock for cropland to grassland land-use change.


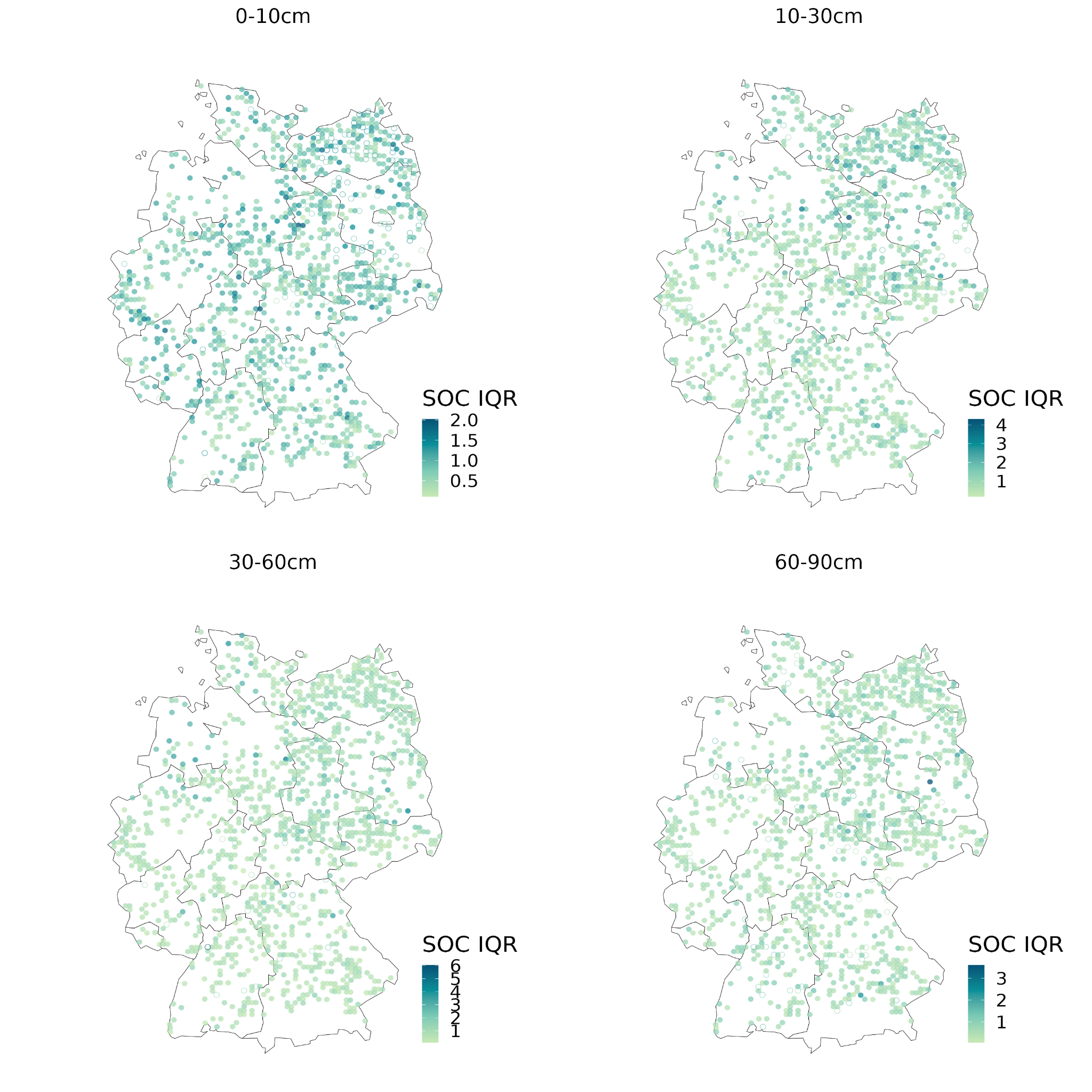


**Figure S7**: Model confidence via interquartile range of model output for the ensemble model predicting SOC stock for grassland to cropland land-use change.


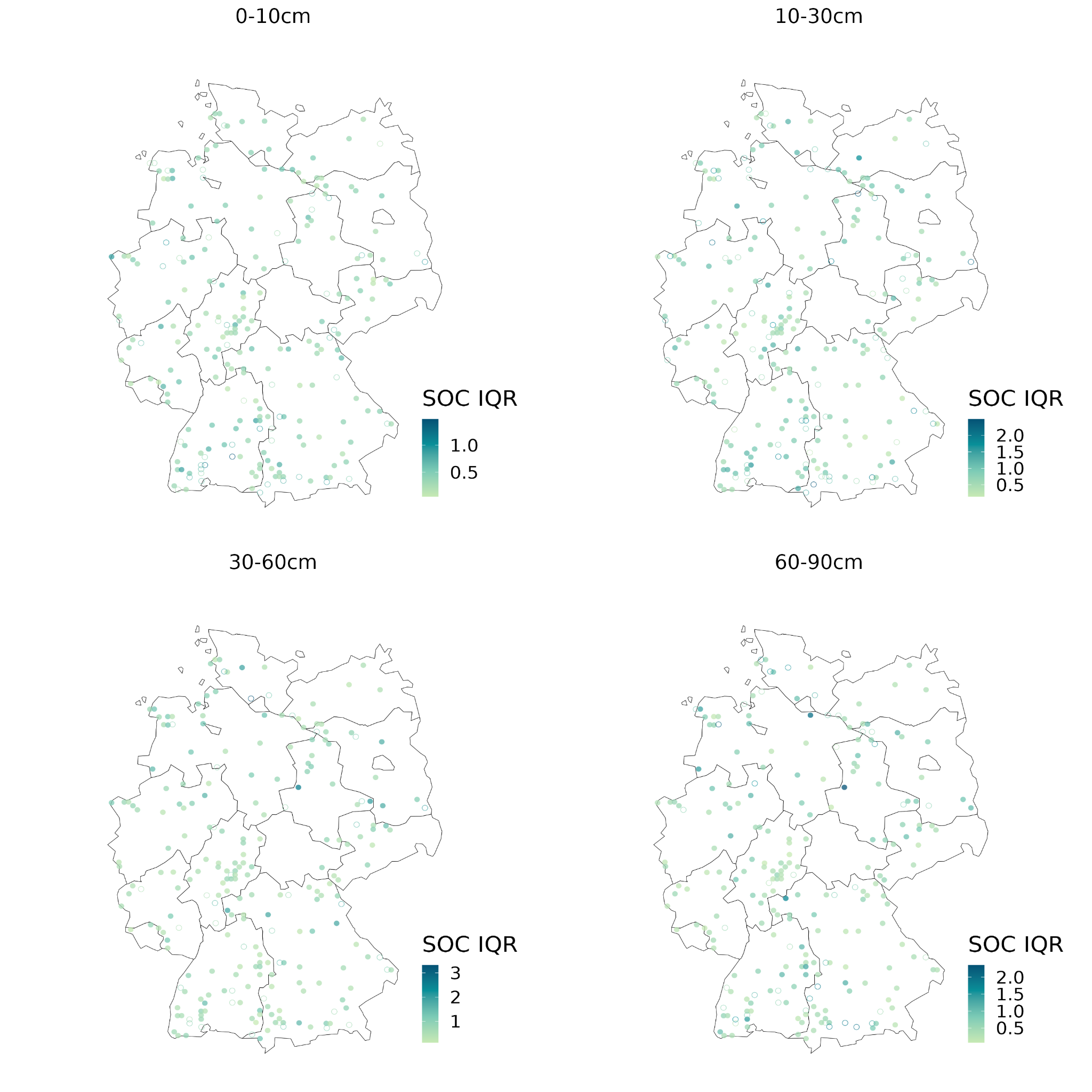


**Figure S8**: Model confidence via interquartile range of model output for the ensemble model predicting SOC stock for cropland to forest land-use change.


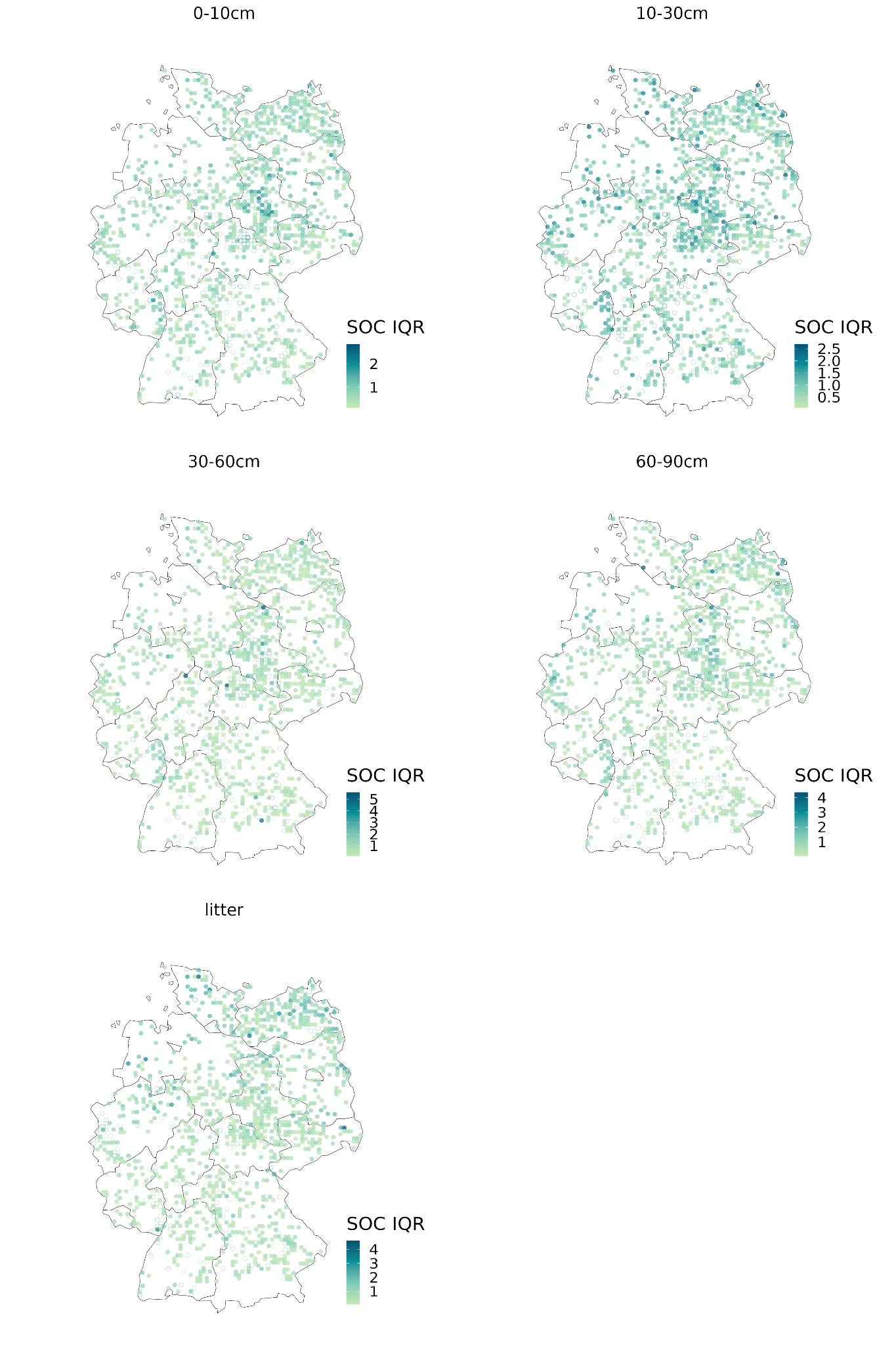


**Figure S9**: Model confidence via interquartile range of model output for the ensemble model predicting SOC stock for forest to cropland land-use change.


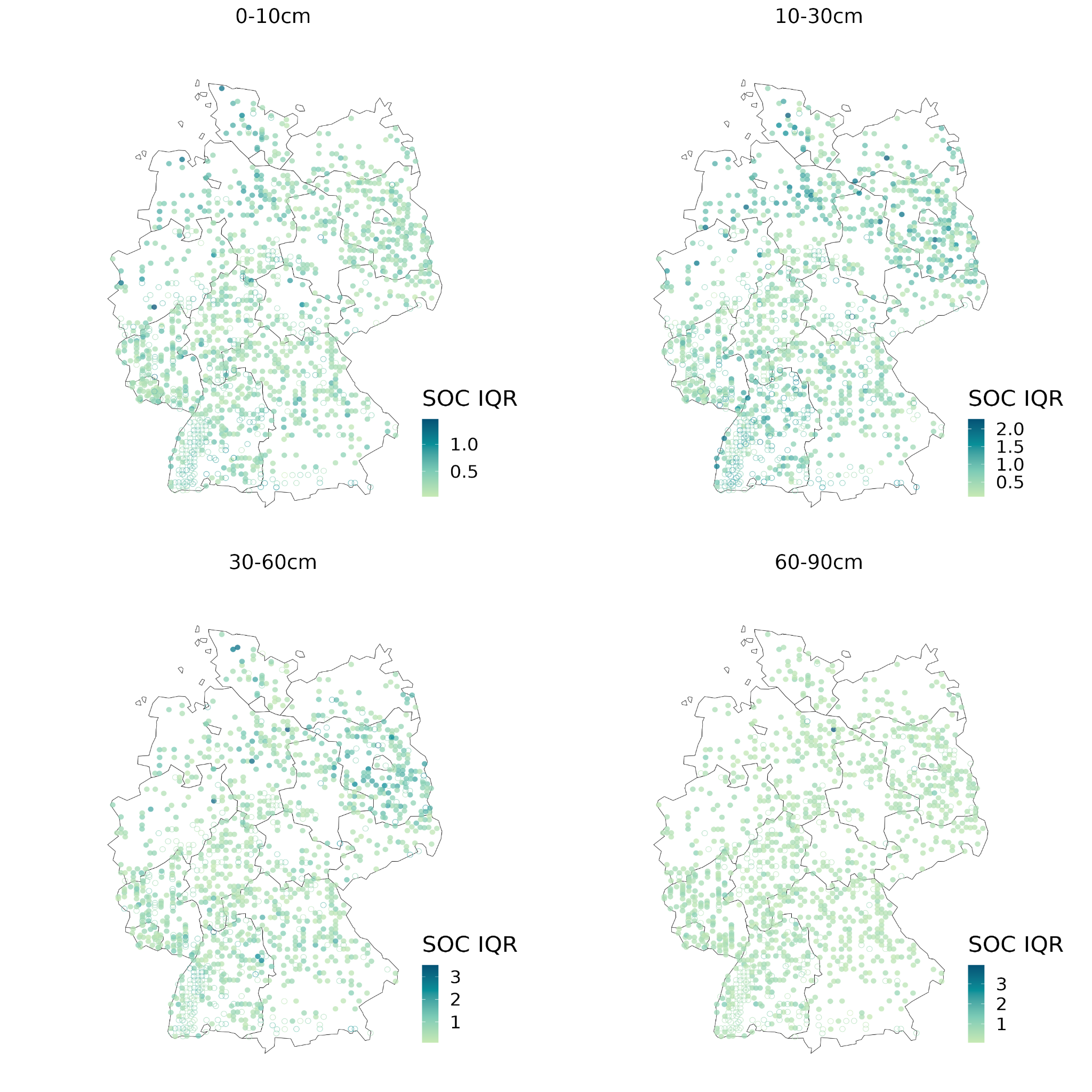


**Figure S10**: Model confidence via interquartile range of model output for the ensemble model predicting SOC stock for grassland to forest land-use change.


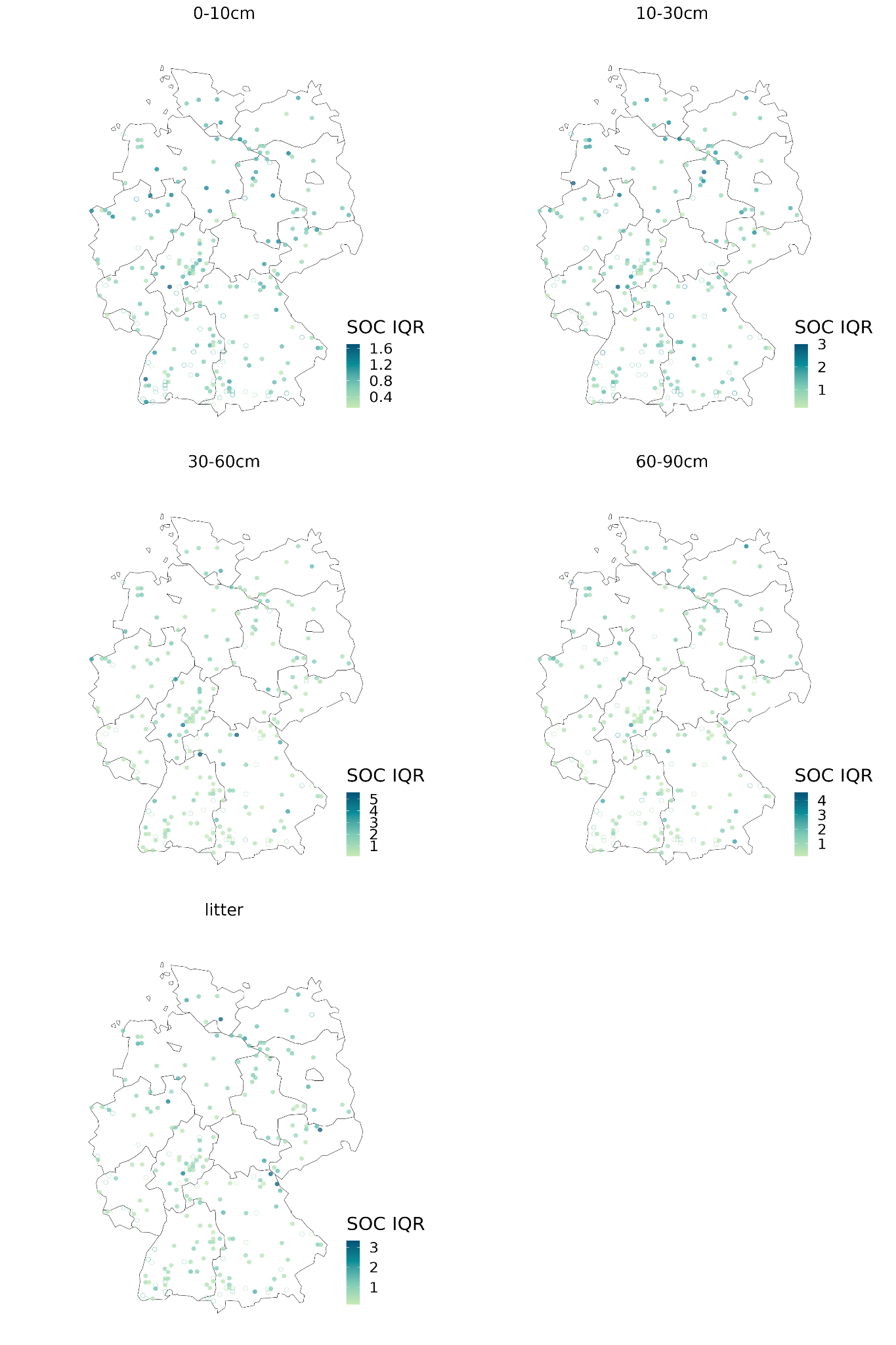


**Figure S11**: Model confidence via interquartile range of model output for the ensemble model predicting SOC stock for forest to grassland land-use change.


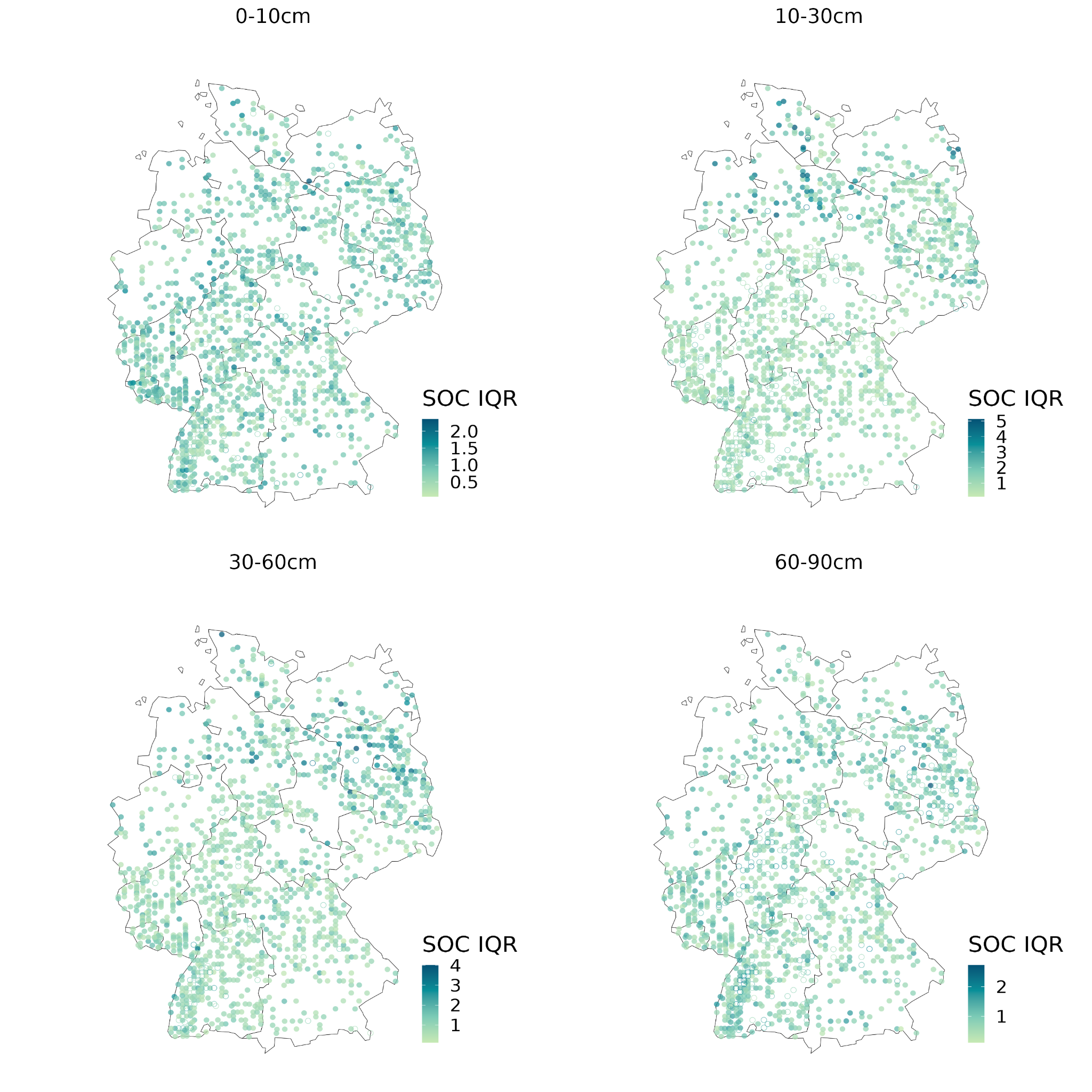


**Figure S12**: Variable importance for SOC stock models for cropland to grassland land-use change (Step 2).


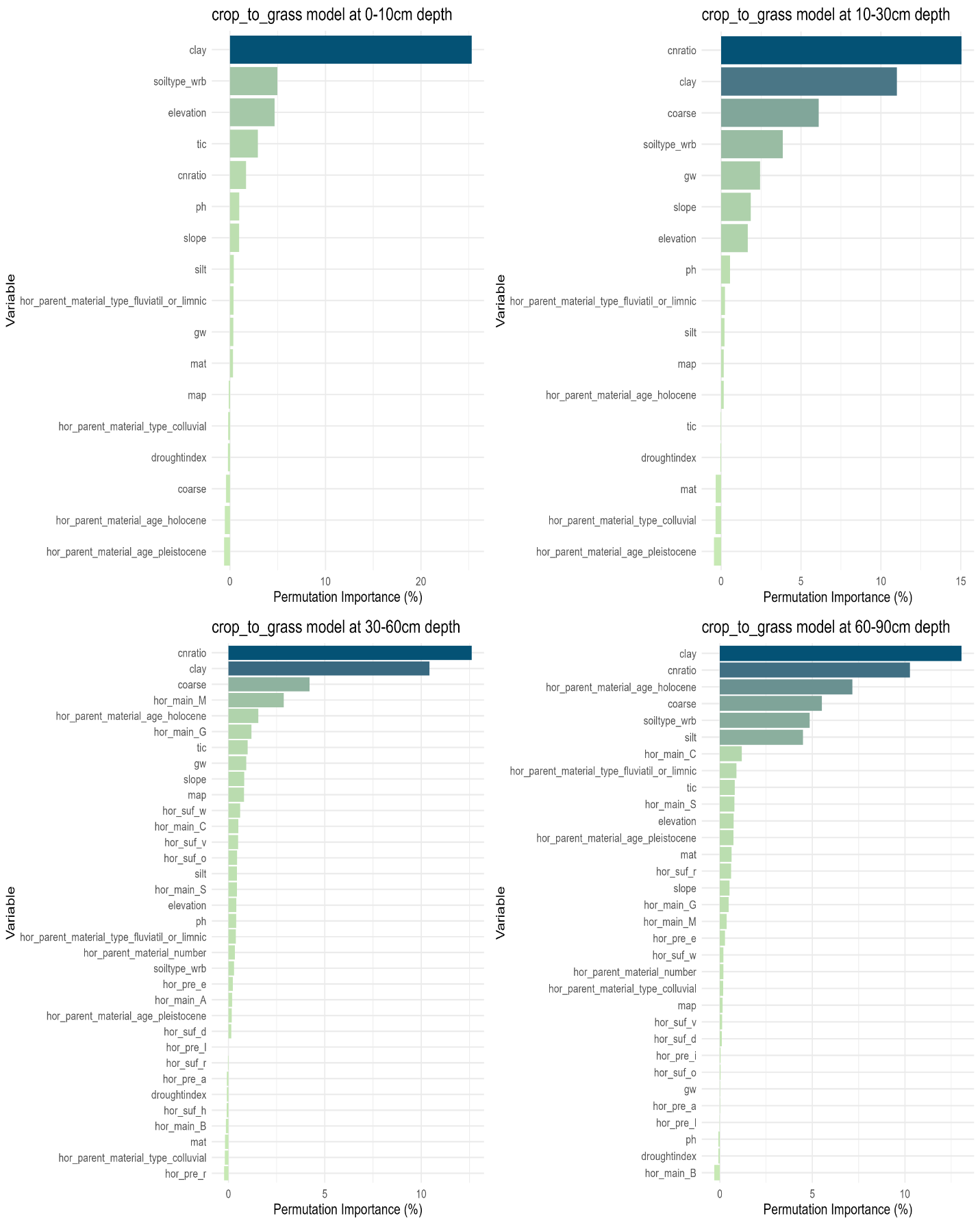


**Figure S13**: Variable importance for SOC stock models for grassland to cropland land-use change (Step 2).


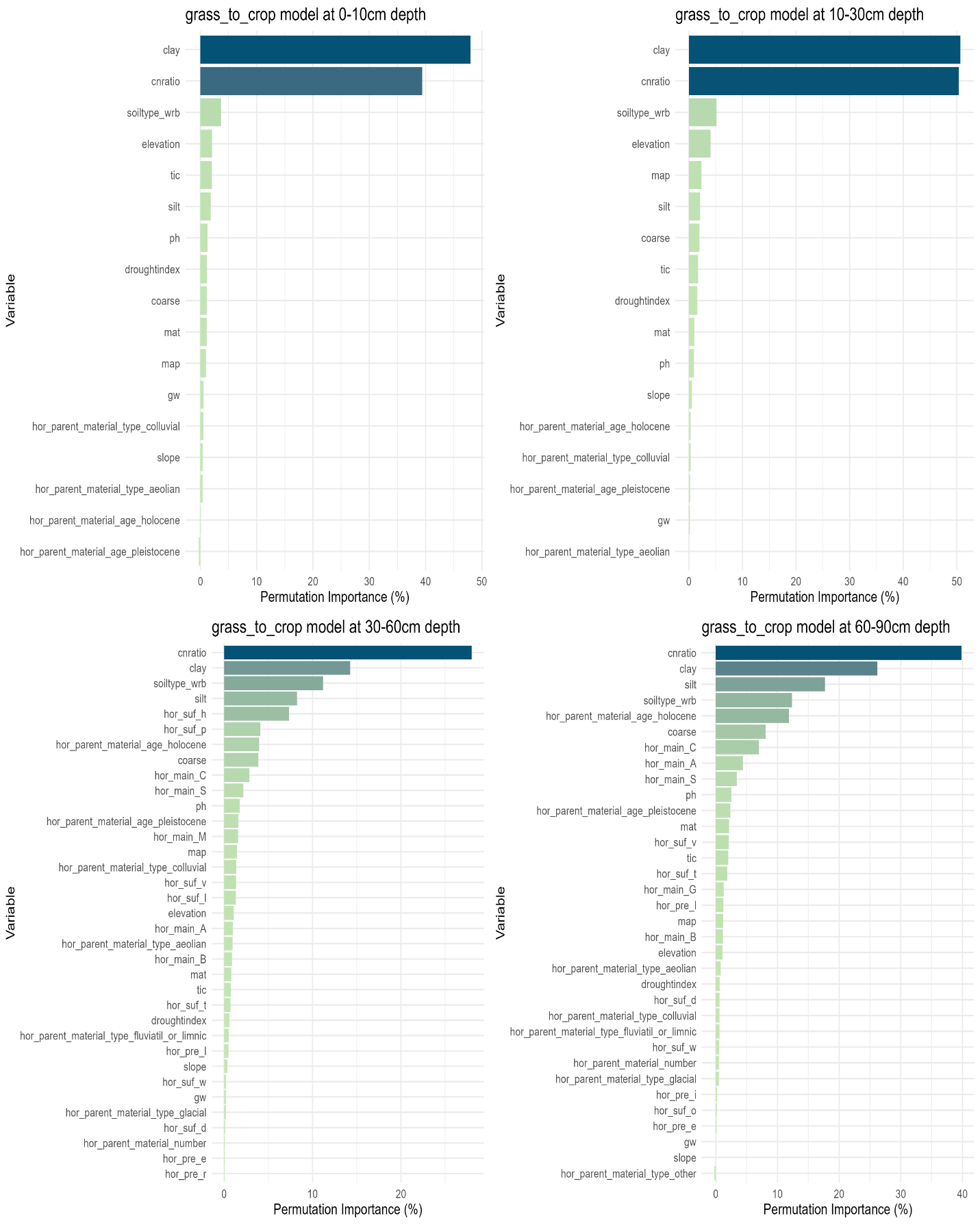


**Figure S14**: Variable importance for SOC stock models for cropland to forest land-use change (Step 2).


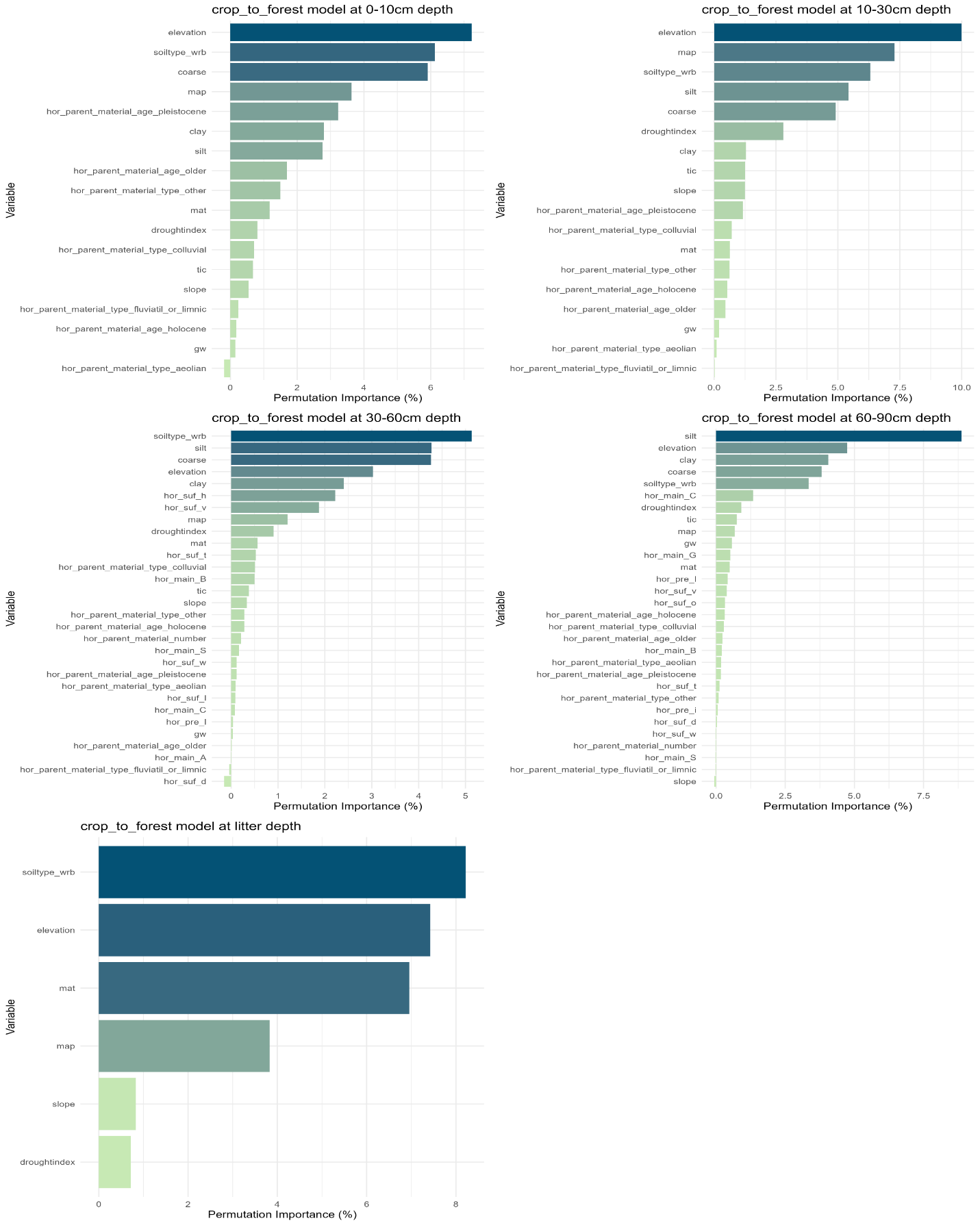


**Figure S15**: Variable importance for SOC stock models for forest to cropland land-use change (Step 2).


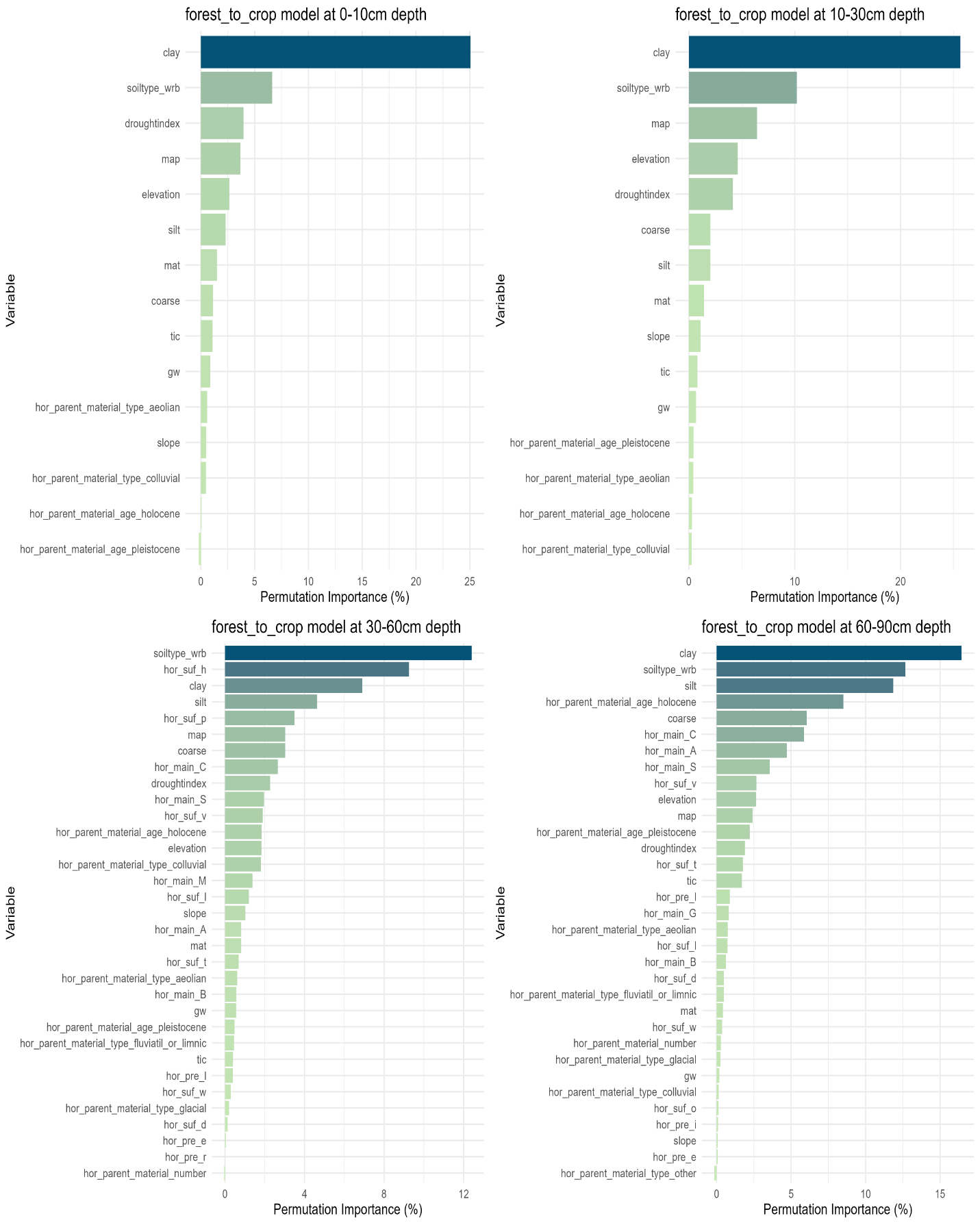


**Figure S16**: Variable importance for SOC stock models for grassland to forest land-use change (Step 2).


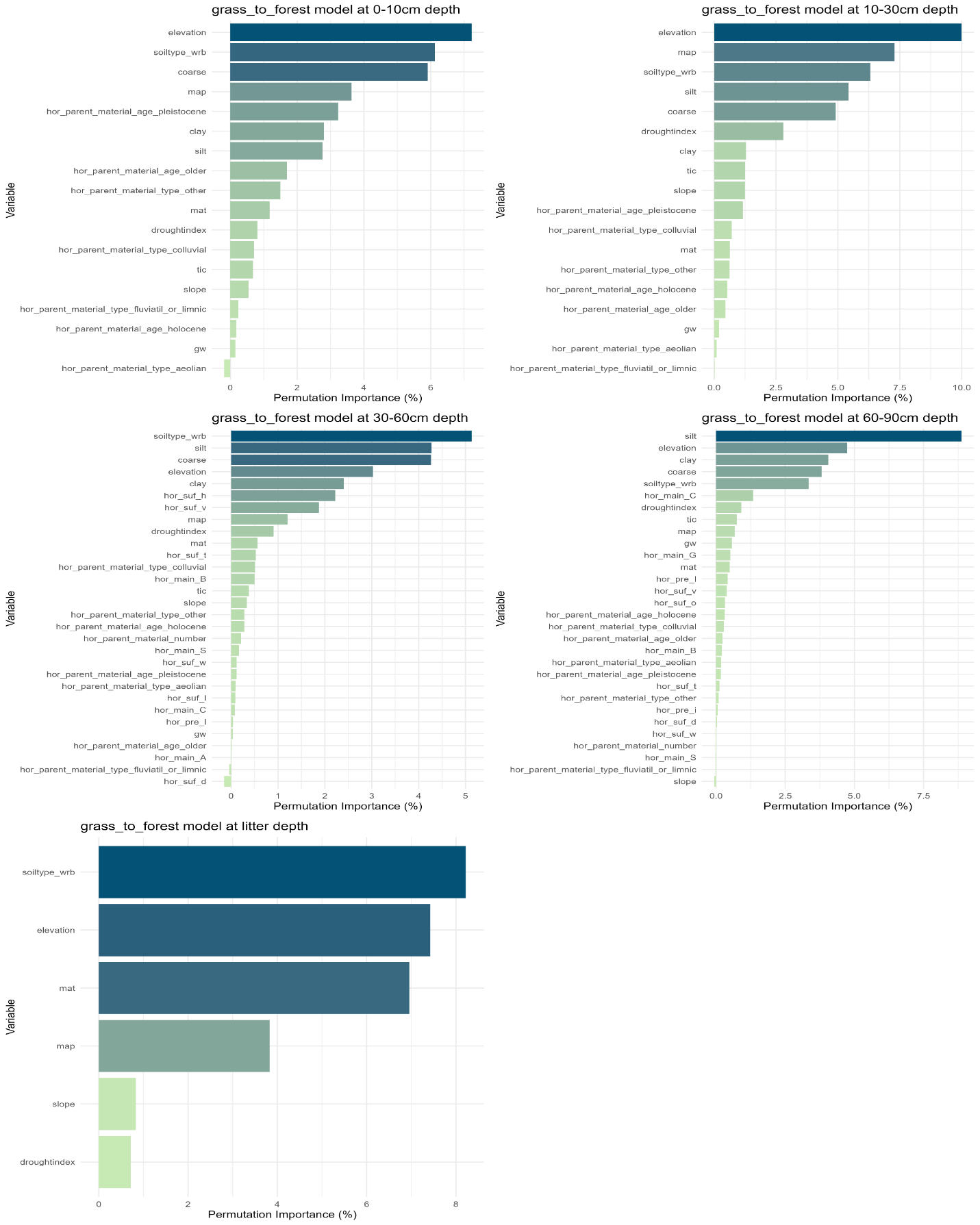


**Figure S17**: Variable importance for SOC stock models for forest to grassland land-use change (Step 2).


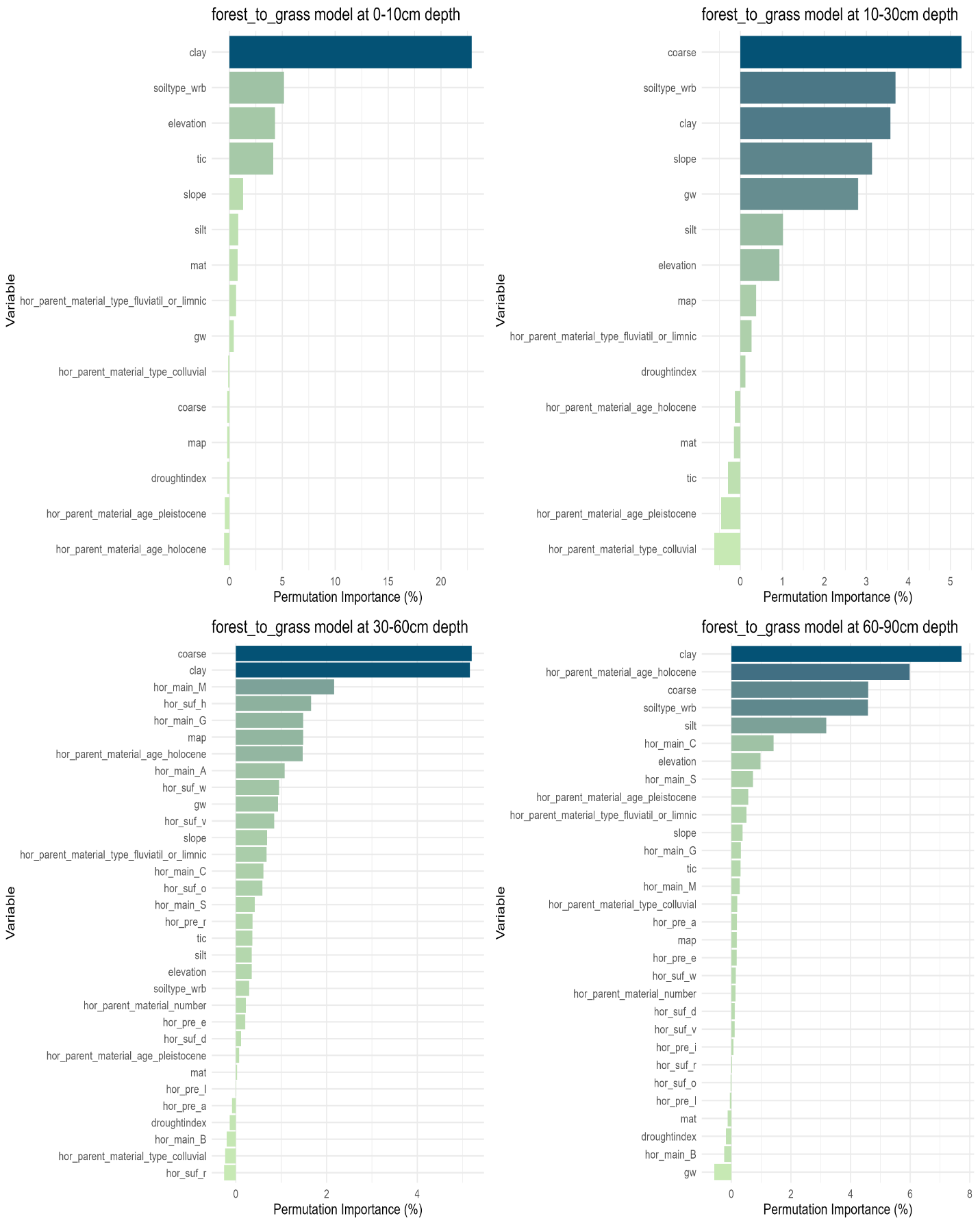


**Figure S18**: ALE for WRB reference soil group for crop <> grass effect size.
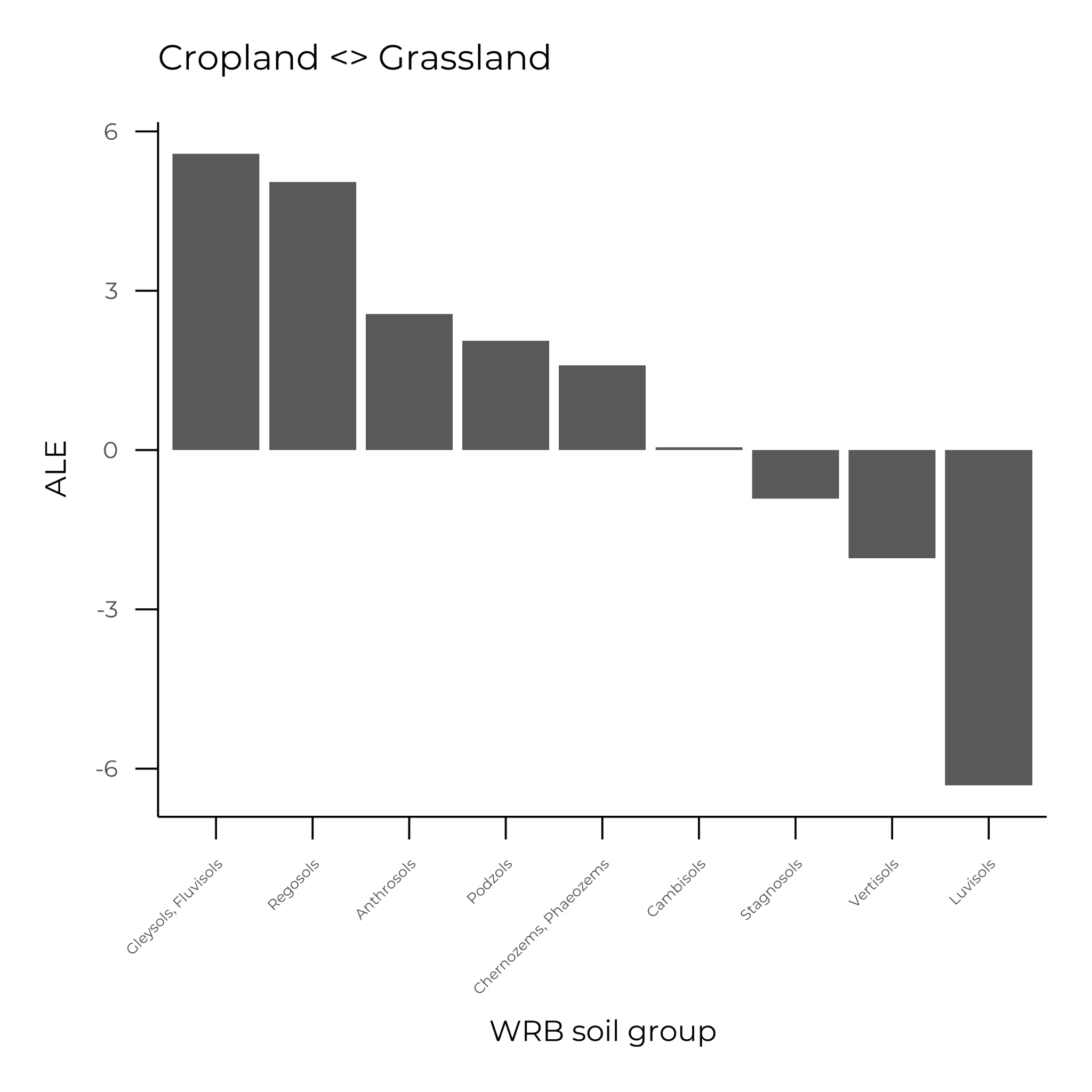


**Figure S19**: ALE for WRB reference soil group for crop <> forest effect size.


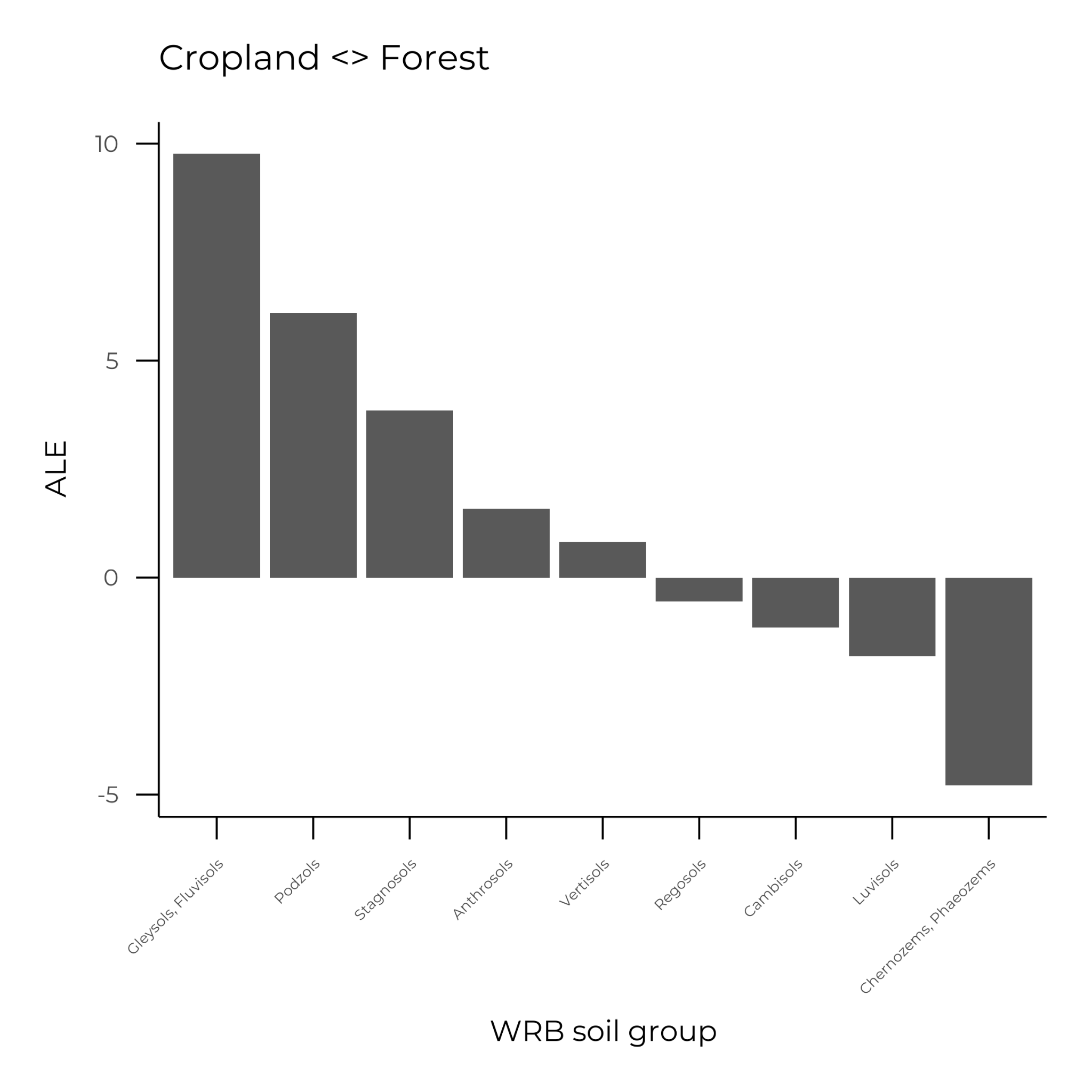


**Figure S20**: ALE for WRB reference soil group for grass <> forest effect size.


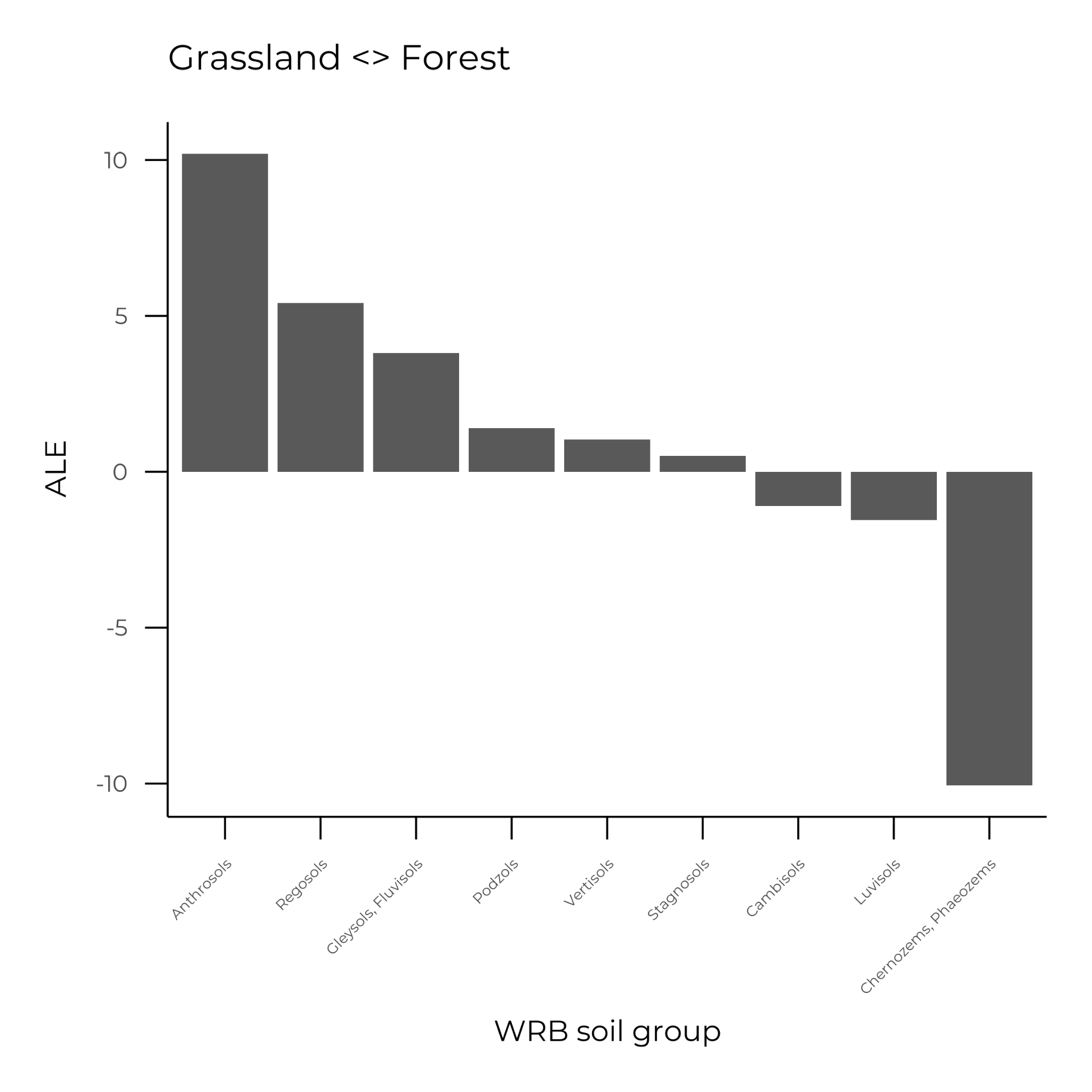

Supplement: Supplementary file 1 — Table S1: Variables used in each model with sources. Figure S1: Distribution of numeric variables by land‐use type for the 0–10 cm depth increment. Figure S2: Distribution of numeric variables by land‐use type for the 10–30 cm depth increment. Figure S3: Distribution of numeric variables by land‐use type for the 30–60 cm depth increment. Figure S4: Distribution of numeric variables by land‐use type for the 60–90 cm depth increment. Figure S5: Distribution of numeric variables by land‐use type for the litter layer. Table S2: Performance metrics for all SOC models produced during Step 2 of the data‐driven reciprocal modeling pipeline. Figure S6: Model confidence via interquartile range of model output for the ensemble model predicting SOC stock for cropland to grassland land‐use change. Figure S7: Model confidence via interquartile range of model output for the ensemble model predicting SOC stock for grassland to cropland land‐use change. Figure S8: Model confidence via interquartile range of model output for the ensemble model predicting SOC stock for cropland to forest land‐use change. Figure S9: Model confidence via interquartile range of model output for the ensemble model predicting SOC stock for forest to cropland land‐use change. Figure S10: Model confidence via interquartile range of model output for the ensemble model predicting SOC stock for grassland to forest land‐use change. Figure S11: Model confidence via interquartile range of model output for the ensemble model predicting SOC stock for forest to grassland land‐use change. Figure S12: Variable importance for SOC stock models for cropland to grassland land‐use change (Step 2). Figure S13: Variable importance for SOC stock models for grassland to cropland land‐use change (Step 2). Figure S14: Variable importance for SOC stock models for cropland to forest land‐use change (Step 2). Figure S15: Variable importance for SOC stock models for forest to cropland land‐use change (Step 2). Figure S16: Variable im [file GCB-31-e70576-s001.docx]
